# DSS-induced colitis is associated with adipose tissue dysfunction and disrupted hepatic lipid metabolism leading to hepatosteatosis and dyslipidemia in mice

**DOI:** 10.1038/s41598-021-84761-1

**Published:** 2021-03-05

**Authors:** Jeonghyeon Kwon, Chungho Lee, Sungbaek Heo, Bobae Kim, Chang-Kee Hyun

**Affiliations:** School of Life Science, Handong Global University, Pohang, Gyungbuk 37554 South Korea

**Keywords:** Chronic inflammation, Inflammatory bowel disease, Non-alcoholic fatty liver disease, Dyslipidaemias, Homeostasis

## Abstract

Considering high prevalence of non-alcoholic fatty liver diseases (NAFLD) in patients with inflammatory bowel disease (IBD), this study aimed to elucidate molecular mechanisms for how intestinal inflammatory conditions are causally linked to hepatic steatosis and dyslipidemia. Both younger and older mice treated with acute or chronic dextran sodium sulfate (DSS) developed colitis, which was evidenced by weight loss, colon length shortening, and elevated disease activity index and inflammation score. They also showed decreased expression of intestinal barrier function-related proteins and elevated plasma lipopolysaccharide level, indicating DSS-induced barrier dysfunction and thereby increased permeability. Interestingly, they displayed phenotypes of hepatic fat accumulation and abnormal blood lipid profiles. This DSS-induced colitis-associated lipid metabolic dysfunction was due to overall disruption of metabolic processes including fatty acid oxidation, lipogenesis, lipolysis, reverse cholesterol transport, bile acid synthesis, and white adipose tissue browning and brown adipose tissue thermogenesis, most of which are mediated by key regulators of energy homeostasis such as FGF21, adiponectin, and irisin, via SIRT1/PGC-1α- and LXRα-dependent pathways. Our study suggests a potential molecular mechanism underlying the comorbidity of NAFLD and IBD, which could provide a key to understanding how the two diseases are pathogenically linked and discovering critical therapeutic targets for their treatment.

## Introduction

Inflammatory bowel disease (IBD), comprised of Crohn’s disease (CD) and ulcerative colitis (UC), are characterized by chronic inflammation associated with intestinal immune dysregulation and impaired epithelial barrier function^1^. Various murine models of chronic intestinal inflammation have been developed to elucidate the pathogenesis of IBD, among which dextran sodium sulfate (DSS)-induced colitis is the most extensively used chemical-induced model due to its simplicity and human ulcerative colitis-like pathologies^2,3^. Although the underlying pathological mechanism in DSS-induced colitis mice is still unclear, it is likely to be multifactorial including the damage of epithelial cells lining the colon, leading to the dissemination of pro-inflammatory intestinal contents into underlying tissue^4^.

The disruption of intestinal epithelium integrity is thought to be an early event in IBD pathogenesis, allowing bacteria and their products to penetrate through a barrier leak, leading to aberrant immune response and inflammation^5^. Altered expression of proteins regulating tight junctions, including occludin, claudins, and zonula occludens (ZOs), could be postulated as a cause of increased intestinal permeability, which also has been observed in IBD patients^6^. Alterations in mucin expression also lead to increased susceptibility to IBD since mucosal layer covering intestinal epithelium serves as a defensive barrier and a reservoir for antimicrobial molecules^7^. In recent years, it has been found that there are interactions between IBD and metabolic disorders, and their pathology shares common features, including metabolic tissue dysregulation, inadequate immune response, and inflammation^8^. However, the pathogenic association and the possible pathophysiologic connections between these two categories of diseases remain largely undescribed^9–14^. Although the concomitance of non-alcoholic fatty liver diseases (NAFLD) among patients with IBD have also been frequently reported^14^, the coexistence of NAFLD and IBD appears to be complicatedly influenced by disease-specific risk factors such as metabolic syndrome, medication-induced hepatotoxicity, and gut microbiota dysbiosis^15,16^. NAFLD has been shown to be associated with an elevated level of circulating endotoxin that activates Kupffer cells to produce pro-inflammatory cytokines leading to hepatocyte damage^17^, which could be a result of impaired intestinal barrier function. However, although multiple factors in the pathogenesis of IBD and NAFLD have been proposed to explain high NAFLD prevalence in IBD patients and both intestinal inflammation and metabolic factors are believed to contribute to the pathogenesis of IBD-associated NAFLD, the causal relationship between these two disorders is still unclear^18,19^.

Basic and clinical studies have demonstrated that NAFLD is associated with changes in some distinct metabolic regulators such as fibroblast growth factor 21 (FGF21), adiponectin, and irisin, which have beneficial effects on energy homeostasis^20–23^. As a common key factor mediating their effects, sirtuin 1 (SIRT1) plays a critical role in lipid and glucose homeostasis, and thus contributes to the pathogenesis of NAFLD^24–28^. It mediates metabolic benefits in major metabolic tissues such as the liver, adipose tissue, and skeletal muscle, through regulating the activity of transcription factors including peroxisome proliferator-activated receptor gamma coactivator 1α (PGC-1α), liver X receptor (LXR), and sterol regulatory element-binding protein (SREBP)^29^. In the liver, PGC-1α is deacetylated and activated by SIRT1, leading to upregulation of genes involved in mitochondrial biogenesis and fatty acid oxidation^30^. SIRT1 also deacetylates LXR to promote not only hepatic cholesterol clearance via upregulation of CYP7A1, a rate-limiting enzyme in bile acid synthesis, but also reverse cholesterol transport (RCT) through upregulating ABCA1 and SR-B1, two primary high-density lipoprotein (HDL)-cholesterol efflux transporters^29,31,32^. Adiponectin signaling through its receptors AdipoR1 and AdipoR2 stimulates AMPK that subsequently activates PGC-1α by direct phosphorylation or SIRT1-mediated deacetylation, resulting in reduced fat accumulation in the liver, skeletal muscle, and adipose tissue^33–35^. Irisin and FGF21 regulate energy metabolism through stimulating white adipose tissue (WAT) browning and potentiating brown adipose tissue (BAT) thermogenic function via PGC-1α-dependent pathways^36^. Bile acids also serve as signaling molecules that regulate energy homeostasis^37^. Bile acid-dependent activation of G protein-coupled bile acid receptor 1 (TGR5) improves glucose homeostasis by inducing glucagon-like peptide-1 (GLP-1) secretion from intestine^38^ and promotes energy expenditure by stimulating mitochondrial biogenesis in BAT^39^.

In the present study, we explored the mechanisms driving the development of NAFLD-like phenotype in DSS-induced colitis model mice, which displayed hepatic steatosis and dyslipidemia. Our focus was on the colitis-associated alterations in lipid metabolism in the liver and adipose tissue, cholesterol and bile acid metabolism in the liver, browning and thermogenesis in adipose tissue. We found that the increased intestinal permeability and consequent endotoxemia caused by colitis were associated with hepatic inflammation and adipose tissue dysfunctions, thereby disrupting hepatic lipid and bile acid metabolism, leading to excessive hepatic fat accumulation and abnormal lipid profile. Given that there are potential interactions between IBD and NAFLD, the elucidation of the detailed molecular mechanisms for how these two diseases are causally linked is critical for the intervention on risk factors to reduce the prevalence of NAFLD comorbidities in IBD. Our findings suggest that IBD-associated metabolic dysfunctions in the liver and adipose tissue could be the causal relationship between IBD and metabolic disorders and regarded as a potential target in the development of therapeutic approaches for their prevention and treatment.

## Results

### DSS-induced colitis mice display tight junction disruption and chronic inflammation in the colon

**Figure 1:**
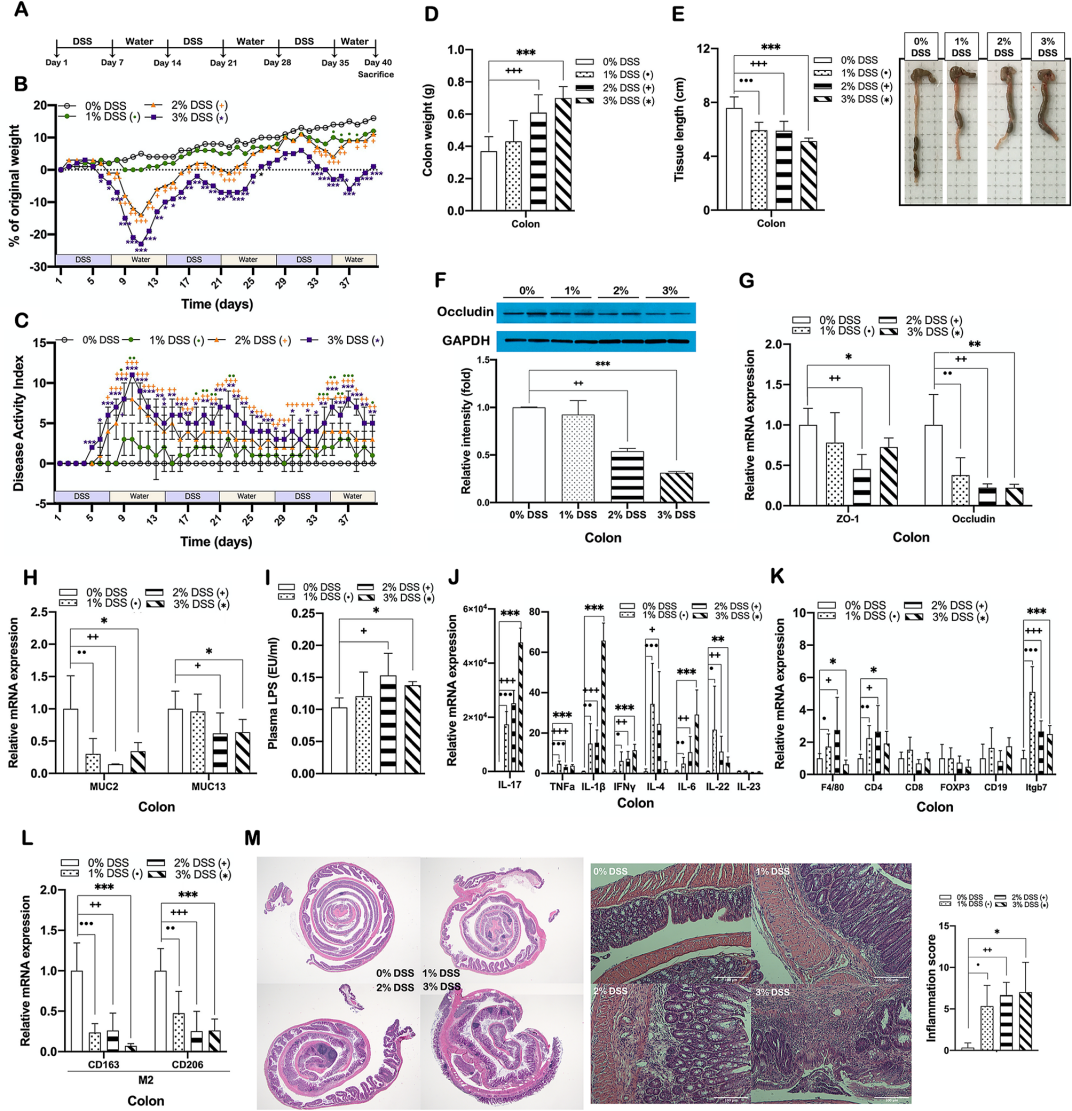
DSS-induced colitis mice display tight junction disruption and chronic inflammation in the colon. (**A**) Experimental scheme illustrating the cycles of DSS and water treatment. Changes in (**B**) body weight and (**C**) disease activity index (DAI) during 3 cycles of DSS treatment. (**D**) Colon weight and (**E**) colon length and representative photographs for colon tissue of DSS-treated and non-treated mice. Protein expression of (**F**) occludin, and mRNA expression of (**G**) tight junction proteins, (**H**) mucins, (**J**) inflammatory cytokines, (**K**) immune cell markers, and (**L**) M2 macrophage markers in the colon of DSS-treated and non-treated mice. Gene expression levels are normalized to GAPDH expression. (**I**) Plasma LPS level in DSS-treated and non-treated mice. (**M**) Representative images of H&E staining of Swiss roll section of colon segment (left; at ×12.5 magnification) and colon tissue (center; ×200; scale bar, 100 μm), and a graph representing inflammation score (right). Data present mean ± SD for 5–8 mice in each group. Statistical significance was determined using two-way ANOVA with (**B**,**C**) with Dunnett’s comparison test. ^•,+,^*p < 0.0332, ^••,++,^**p < 0.0021, ^•••,+++,^***p < 0.0002. Student’s two-tailed t-test was used for the analysis of differences between experimental groups (**D**–**M**). ^•,+,^*p < 0.05, ^••,++,^**p < 0.01, ^•••,+++,^***p < 0.001. 0% DSS, non-DSS-treated group; 1, 2, 3% DSS, 1, 2, and 3% (w/v) DSS-treated groups, respectively.

Mice treated with DSS at concentrations of 1, 2, and 3% in drinking water for 7 days followed by 7 days of recovery with normal drinking water (Fig. 1A) developed colitis, as indicated by significant decreases in body weight (Fig. 1B) and significant increases in the severity of colitis assessed by disease activity index (DAI) scoring compared to non-DSS treated control mice (Fig. 1C). This was accompanied by significant reductions in colon weight and increases in colon length (Fig. 1D,E). DSS-treated mice also had significantly decreased expression of genes related to tight junction function and mucus layer formation (Fig. 1F–H), and significantly increased plasma lipopolysaccharide (LPS) level (Fig. 1I) compared to non-DSS treated control mice. In addition, the colonic expressions of pan-macrophage marker (F4/80), T helper cell marker (CD4), and gut mucosal homing marker integrin β7 (Itgb7), as well as pro-inflammatory cytokines including IL-17, TNFα, IL-1β, IFNγ, IL-4, and IL-6 (Fig. 1J,K) were significantly upregulated, while expression of anti-inflammatory M2 macrophage markers (CD163 and CD206) was significantly downregulated (Fig. 1L), indicating that DSS treatment induced macrophage infiltration and pro-inflammatory responses in the colon. Increased colonic inflammation was also confirmed by histological analysis that showed significant transmural inflammation in colon sections of DSS-treated mice, which was reflected by inflammation score, with marked increase in crypt damage, immune cell infiltration into lamina propria, and alteration of epithelial and mucosal structure, compared to non-DSS treated mice (Fig. 1M).

### DSS-induced colitis is associated with hepatic steatosis in mice

**Figure 2:**
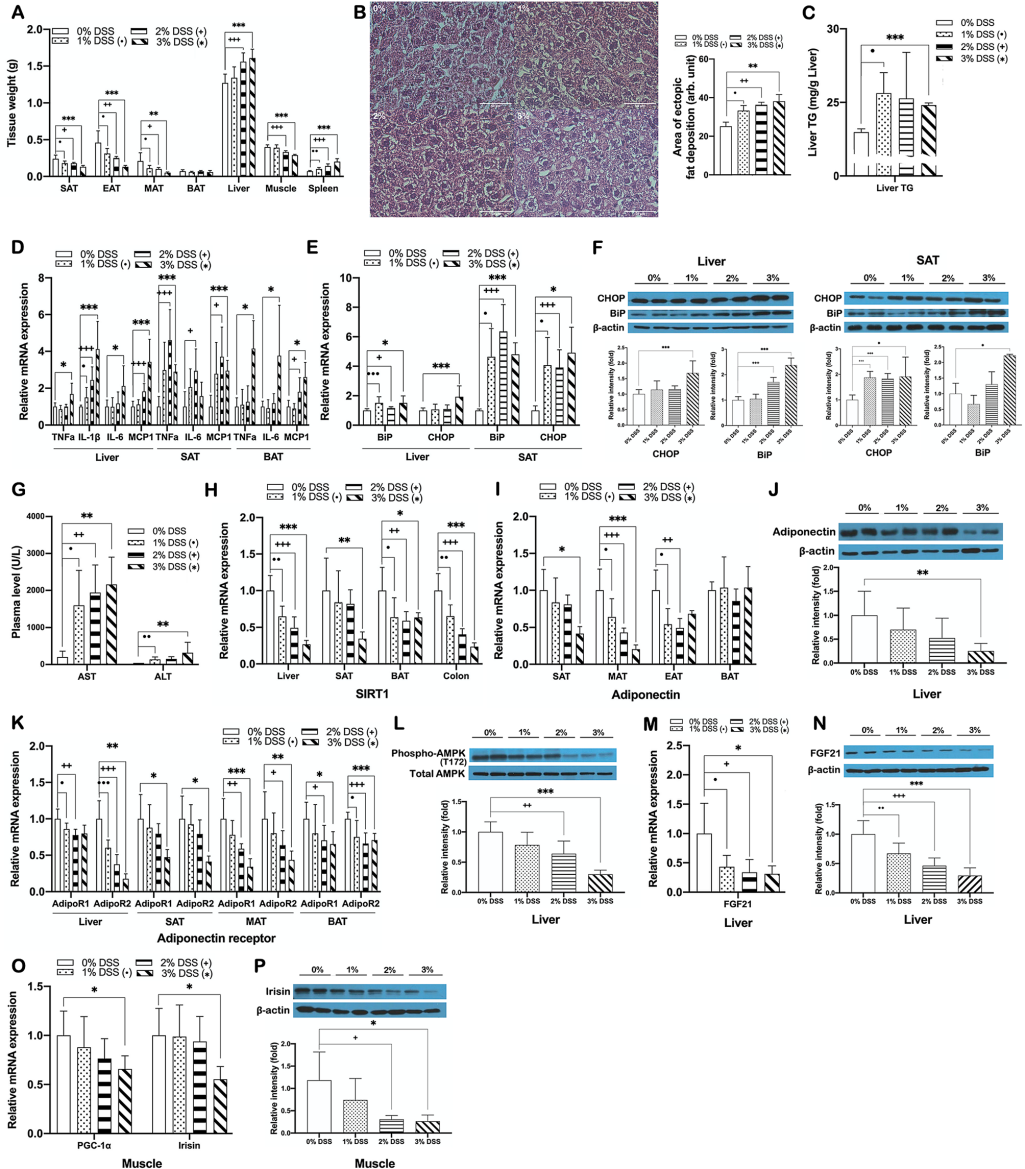
DSS-induced colitis is associated with hepatic steatosis in mice. (**A**) Tissue weights of DSS-induced colitis and non-colitis mice (**B**) Representative images of H&E staining of liver sections (left, ×400; scale bar, 50 μm) and a graph presenting area of ectopic fat deposition (right). (**C**) Hepatic TG content. mRNA expression of (**D**) pro-inflammatory cytokines in the liver, SAT, and BAT, and (**E**) ER stress-related markers in the liver and SAT, (**H**) SIRT1 in the liver, SAT, BAT, and the colon, (**I**) adiponectin in adipose tissues, (**K**) adiponectin receptors in the liver and adipose tissues, (**M**) FGF21 in the liver, and (**O**) PGC-1α and irisin in skeletal muscle of DSS-induced colitis and non-colitis mice. Gene expression levels are normalized to GAPDH or Arbp expression. Protein expression of (**F**) CHOP and BiP in the liver and SAT, (**J**) adiponectin in SAT, (**L**) phosphorylated and total AMPK in the liver, (**N**) FGF21 in the liver, and (**P**) irisin in skeletal muscle of DSS-treated and non-treated mice. (**G**) Plasma levels of AST and ALT in DSS-induced colitis and non-colitis mice. Data present mean ± SD for 5–8 mice in each group. Student’s two-tailed t-test was used for the analysis of differences between experimental groups. ^•,+,^*p < 0.05, ^••,++,^**p < 0.01, ^•••,+++,^***p < 0.001. SAT, MAT, EAT, and BAT, subcutaneous, mesenteric, epididymal, and brown adipose tissue, respectively.

In parallel to a significant body weight loss, DSS-induced colitis mice showed a significant reduction in the weight of WATs including epididymal, mesenteric, and subcutaneous adipose tissues (EAT, MAT, and SAT) (Fig. 2A). In contrast, interestingly, a significant increase in liver weight was observed in DSS-treated mice compared to non-DSS treated controls, which was confirmed by histological observation of significant increases in ectopic fat deposition and triglyceride (TG) content in the liver (Fig. 2B,C). There were also significant increases in expression of pro-inflammatory cytokines such as TNFα, IL-1β, IL-6 and MCP1 in the liver, SAT, and BAT (Fig. 2D) and ER stress-related proteins in the liver and SAT (Fig. 2E,F). Besides, it was also observed that the biochemical parameters of liver damage such as plasma levels of alanine aminotransferase (ALT) and aspartate aminotransferase (AST) were significantly increased in DSS-induced colitis mice (Fig. 2G).

To investigate how DSS-induced colitis is associated with hepatic steatosis, we first searched for the molecular pathways that might play pivotal roles in the pathogenesis of the two disorders. We observed that the expression of SIRT1, known as a master metabolic regulator, was significantly decreased in several tissues including the liver, SAT, BAT, and colon of DSS-induced colitis mice compared to non-colitis control mice (Fig. 2H). Adiponectin, an adipokine closely related to activation of SIRT1^33^, was also observed to be significantly lower in adipose tissues, including SAT, MAT, and EAT, of DSS-induced colitis mice than those in non-colitis controls (Fig. 2I,J). Along with the reduction in adiponectin expression, the expression level of adiponectin receptors in the liver, SAT, MAT, and BAT (Fig. 2K) and the phosphorylation level of AMPK at Thr-172 in the liver were also significantly reduced in DSS-induced colitis mice (Fig. 2L). In addition, given the fact that defective adaptive thermogenesis is also one factor that is related to an increased risk of hepatic steatosis^40^, we examined changes in expression of key mediators of thermogenic program including FGF21 and irisin in the liver and skeletal muscle, respectively. DSS-induced colitis mice showed a significant reduction in expression levels of both hepatic FGF21 and skeletal muscle irisin compared to non-colitis control mice (Fig. 2M–P). These data together suggested that DSS-induced colitis was associated with hepatic steatosis, which was possibly caused by, at least in part, suppressed expression of key metabolic regulators including SIRT1, adiponectin, FGF21, and irisin.

### DSS-induced colitis disrupts lipid metabolism in the liver and adipose tissue causing hepatic fat accumulation and dyslipidemia

**Figure 3:**
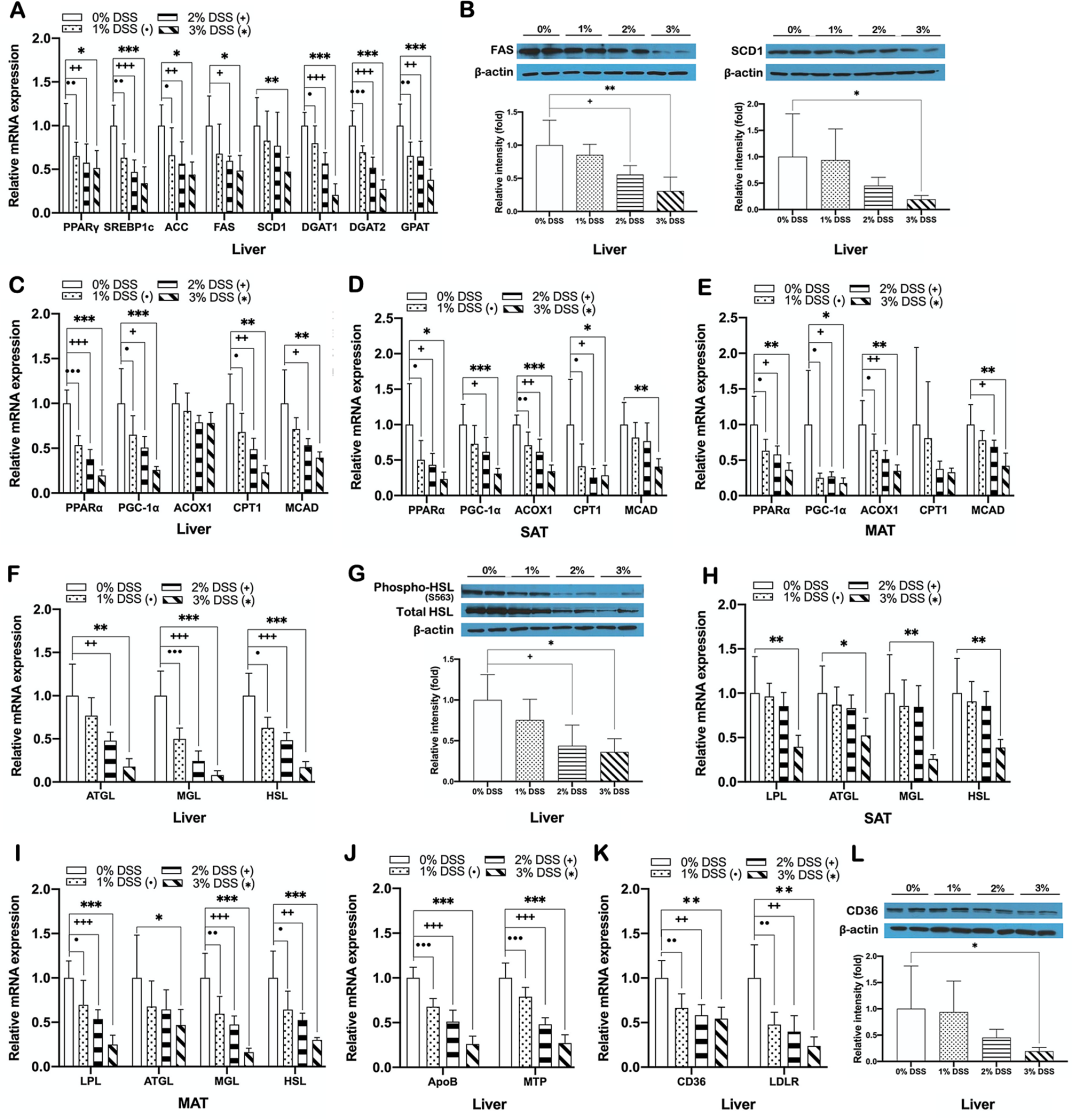
DSS-induced colitis disrupts lipid metabolism in the liver and adipose tissue causing hepatic fat accumulation and dyslipidemia. mRNA expression of (**A**) lipogenic genes in the liver, (**C**–**E**) fatty acid oxidation genes in the liver, SAT, and MAT, (**F**,**H**,**I**) lipolytic genes in the liver, SAT, and MAT, (**J**) ApoB and MTP in the liver, and (**K**) fatty acid uptake genes in the liver of DSS-induced colitis and non-colitis mice. Gene expression levels are normalized to GAPDH or Arbp expression. Protein expression of (**B**) FAS and SCD1, (**G**) phosphorylated and total HSL in the liver, and (**L**) CD36 in the liver of DSS-treated and non-treated mice. Data present mean ± SD for 5–8 mice in each group. Student’s two-tailed t-test was used for the analysis of differences between experimental groups. ^•,+,^*p < 0.05, ^••,++,^**p < 0.01, ^•••,+++,^***p < 0.001.

To explore more detailed molecular mechanisms involved in the association between DSS-induced colitis and hepatic steatosis, we first examined changes in the expression of key genes related to lipogenesis, lipid oxidation, and lipolysis in the liver and adipose tissues. Despite significant increases in the liver weight and hepatic fat accumulation as shown in Fig. 2A–C, we unexpectedly observed that the expression of genes involved in de novo lipogenesis such as PPARγ, SREBP1c, ACC, FAS, and SCD1, DGAT1, DGAT2, and GPAT was significantly decreased in the liver of DSS-induced colitis mice compared to non-colitis controls (Fig. 3A). The protein expression of FAS and SCD1, which are important lipogenic enzymes, was also significantly reduced in colitis mice (Fig. 3B). DSS-induced colitis mice also showed significant decreases in expression of genes related to lipid oxidation, including PPARα and PGC-1α, and their target genes Acox1, CPT1, and MCAD, as well as lipolytic genes including LPL, ATGL, MGL, and HSL, in the liver, SAT, and MAT compared to non-colitis control mice (Fig. 3C–F,H,I). The levels of both total and phosphorylated HSL, a major lipolytic protein, were also significantly decreased in colitis mice (Fig. 3G). In addition, hepatic expression of ApoB and MTP, which are required for very-low-density lipoprotein (VLDL) assembly and hepatic TG secretion, and key genes related to fatty acid uptake, including CD36 and LDLR, in DSS-induced colitis mice was also significantly lower than that of non-colitis controls (Fig. 3J,K). The protein expression of CD36, a major player in fatty acid uptake, was also significantly reduced in colitis mice (Fig. 3L). Together, these results indicated that DSS-induced colitis caused a comprehensive and substantial dysfunction of lipid metabolism in the liver and adipose tissue, possibly leading to DSS-induced colitis-associated hepatic steatosis.

### DSS-induced colitis impairs cholesterol and bile acid homeostasis

**Figure 4:**
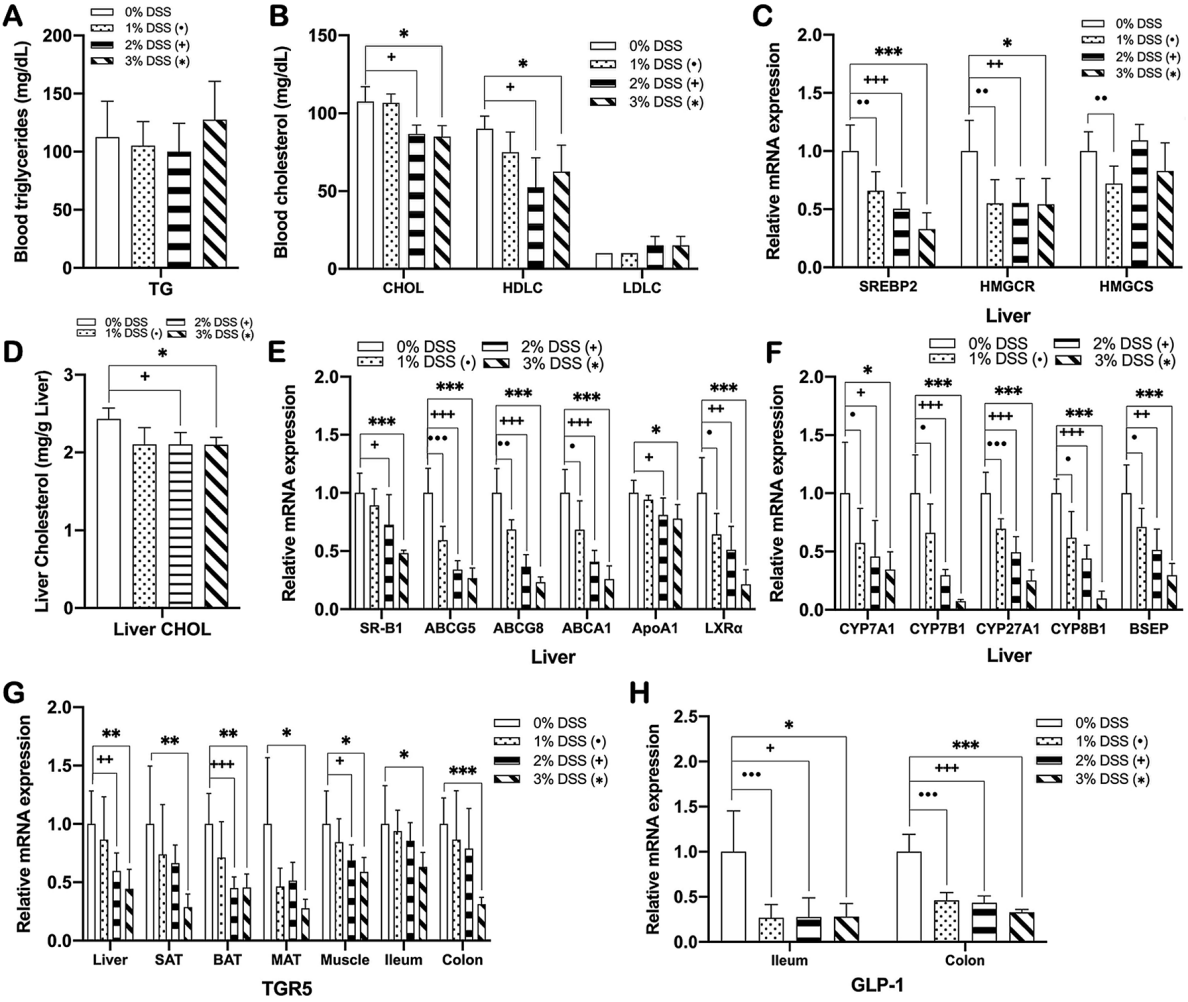
DSS-induced colitis impairs cholesterol and bile acid homeostasis. Blood levels of (**A**) TG, (**B**) total, HDL- and LDL-cholesterol, and (**D**) hepatic level of total cholesterol in DSS-induced colitis and non-colitis mice. mRNA expression of (**C**) cholesterol synthesis genes, (**E**) RCT genes, (**F**) bile acid synthesis genes in the liver, (**G**) bile acid receptor in the liver, SAT, BAT, MAT, skeletal muscle, the ileum, and the colon, and (**H**) GLP-1 in the ileum and the colon of DSS-induced colitis and non-colitis mice. Gene expression levels are normalized to GAPDH or Arbp expression. Data present mean ± SD for 5–8 mice in each group. Student’s two-tailed t-test was used for the analysis of differences between experimental groups. ^•,+,^*p < 0.05, ^••,++,^**p < 0.01, ^•••,+++,^***p < 0.001. CHOL, total cholesterol; HDLC, HDL-cholesterol; LDLC, LDL-cholesterol.

We next tested whether DSS-induced colitis causes an imbalance in cholesterol homeostasis, which is also causally linked to fatty liver disease and dyslipidemia^41^. DSS-induced colitis mice showed a significant reduction in plasma HDL-cholesterol level compared to non-colitis controls, while there was no significant difference in the levels of TG, total cholesterol, and LDL cholesterol between the two groups (Fig. 4A,B). The expression of SREBP2 and cholesterol biosynthetic enzymes including HMGCS and HMGCR (Fig. 4C), and RCT-related genes including SR-B1, ABCG5, ABCG8, ABCA1, ApoA1, and LXRα was significantly downregulated in the liver (Fig. 4E), paralleled with significant decreases in hepatic cholesterol content (Fig. 4D) and plasma HDL-cholesterol level (Fig. 4B), in DSS-induced colitis mice compared to non-colitis controls. Since bile acid synthesis is a major pathway for hepatic cholesterol catabolism and excess cholesterol removal^42^, we further tested changes in the expression of bile acid synthetic genes in response to DSS treatment. The expression levels of genes involved in classical and alternative bile acid synthetic pathways such as CYP7A1, CYP7B1, CYP27A1, and CYP8B1 were significantly lower in the liver of DSS-induced colitis mice than those of non-colitis controls, accompanied with a reduction in expression of bile acid transporter BSEP (Fig. 4F). In addition, the expression of bile acid receptor TGR5 was also significantly downregulated in the liver, SAT, BAT, MAT, muscle, ileum, and colon of DSS-induced colitis mice (Fig. 4F). Consistent with the fact that bile acid-mediated TGR5 activation stimulates the secretion of GLP-1 from enteroendocrine cells^38^, this change was commensurate with decreased GLP-1 expression in ileum and colon of DSS-induced colitis mice (Fig. 4H). DSS-induced colitis mice also showed a significant downregulation in hepatic LXRα expression compared to non-colitis control mice (Fig. 4E). Given the fact that LXRα plays an essential role in regulating cholesterol and bile acid homeostasis^43,44^, this result indicated that DSS-induced colitis-associated reduction in LXRα expression, at least in part, contributed to the development of dyslipidemia.

### DSS-induced colitis suppresses the expression of genes involved in SAT browning and BAT thermogenesis

**Figure 5:**
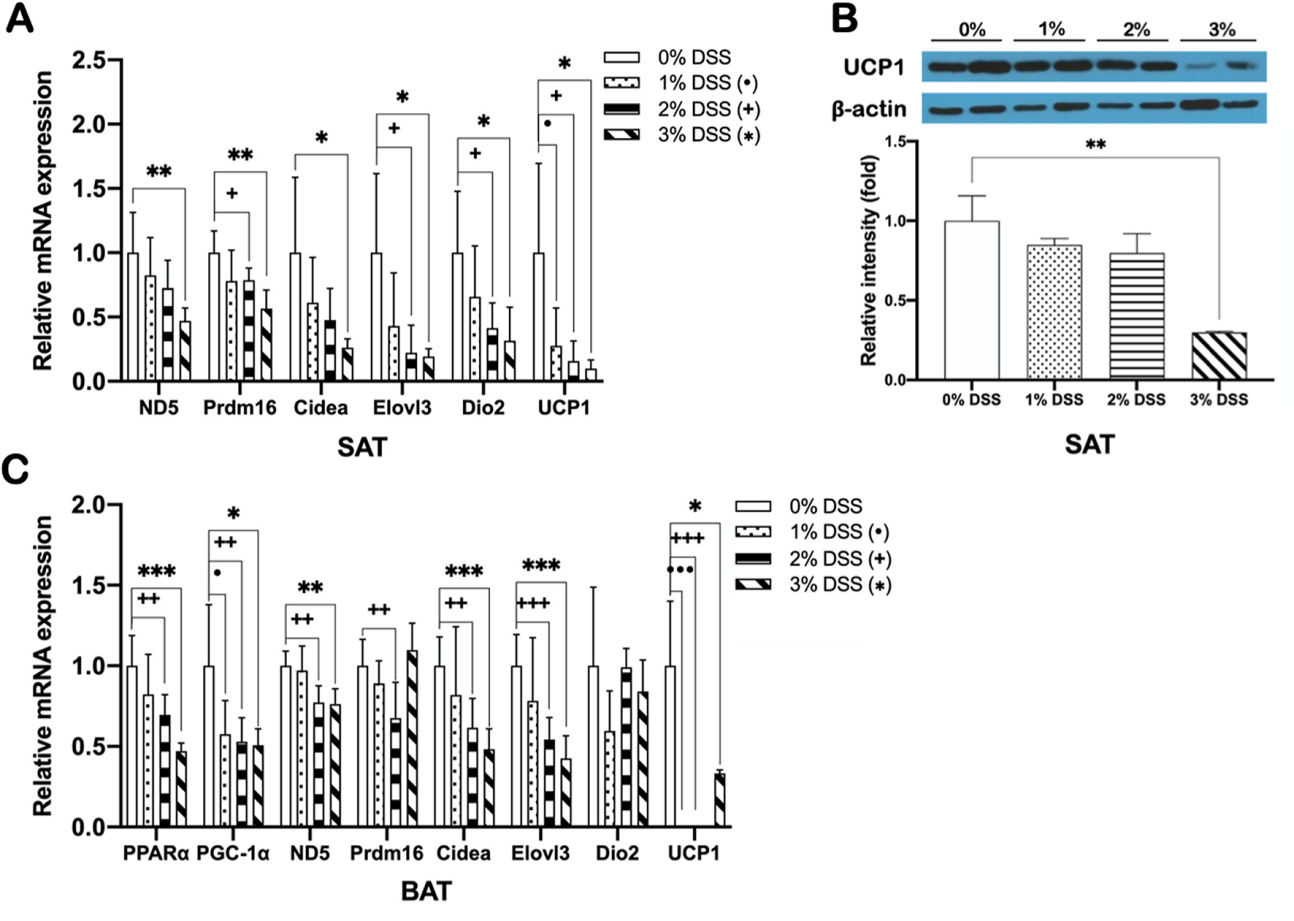
DSS-induced colitis suppresses the expression of genes involved in SAT browning and BAT thermogenesis. mRNA expression of (**A**) browning genes in SAT and (**C**) thermogenic genes in BAT, and protein expression of (**B**) UCP1 in SAT of DSS-induced colitis and non-colitis mice. Gene expression levels are normalized to GAPDH expression. Data present mean ± SD for 5–8 mice in each group. Student’s two-tailed t-test was used for the analysis of differences between experimental groups. ^•,+,^*p < 0.05, ^••,++,^**p < 0.01, ^•••,+++,^***p < 0.001.

To check whether DSS-induced colitis alters the function of adipose tissues thereby reducing energy expenditure and thermogenic capacity, we examined the expression of genes related to SAT browning and BAT thermogenesis. DSS-induced colitis mice showed significantly decreased expressions of PPARα, PGC-1α, and thermogenic genes including ND5, Prdm16, Cidea, Elovl3, Dio2, and UCP1 both in SAT and BAT compared to non-colitis control mice (Fig. 5A,C). The protein expression level of UCP1 was also significantly decreased in SAT of colitis mice (Fig. 5B). Together with the results showing a significant reduction of bile acid synthesis and TGR5 expression (Fig. 4F,G), this data suggested that the increased hepatic fat accumulation of DSS-induced colitis was at least in part caused by decreased bile acid-induced energy expenditure, which was observed as a suppression of TGR5-mediated SAT browning and BAT thermogenesis.

### DSS-induced colitis-associated hepatic steatosis and dyslipidemia are displayed also in older mice

**Figure 6:**
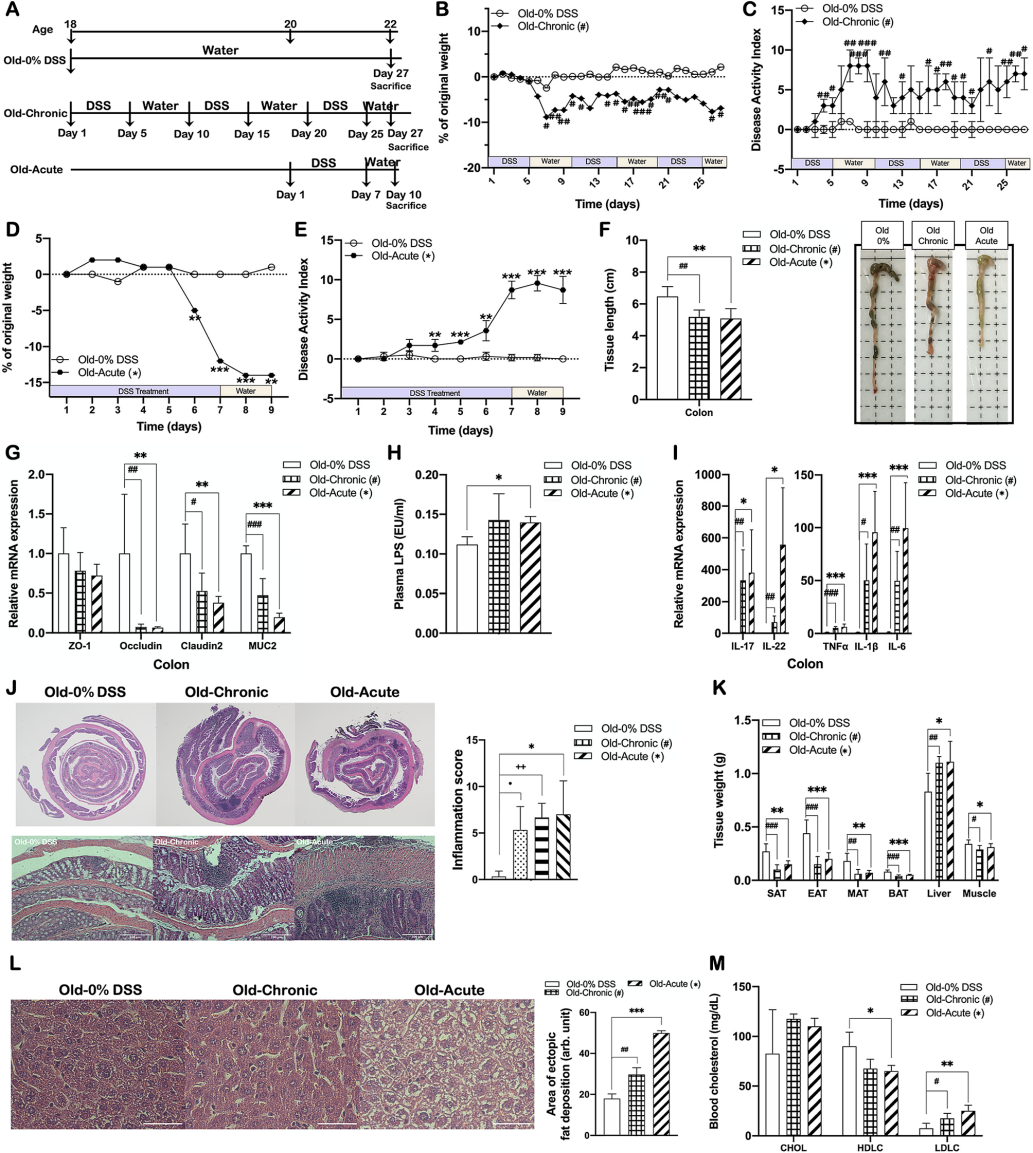
DSS-induced colitis-associated hepatic steatosis and dyslipidemia are displayed also in older mice. (**A**) Experimental scheme illustrating the cycles of DSS and water treatment. Changes in (**B**,**D**) body weight and (**C**,**E**) DAI during chronic and acute DSS treatment, respectively. (**F**) Colon length and representative photographs for colon tissue of DSS-treated and non-treated mice. Colonic expression of (**G**) tight junction proteins and mucin, and (**I**) inflammatory cytokines in DSS-treated and non-treated mice. Gene expression levels are normalized to GAPDH expression. (**J**) Representative images of H&E staining of Swiss roll section of colon segment (left; ×12.5) and colon tissue (center; ×200; scale bar, 100 μm), and a graph representing inflammation score (right). (**K**) Tissue weights of DSS-induced colitis and non-colitis mice. (**L**) Representative images of H&E staining of liver sections (×400; scale bar, 50 μm) and a graph presenting area of ectopic fat deposition. Plasma levels of (H) LPS, (M) total, HDL- and LDL-cholesterol in DSS-induced colitis and non-colitis mice. Data present mean ± SD for 6–7 mice in each group. Statistical significance was determined using two-way ANOVA with (**B**–**E**) with Dunnett’s comparison test. ^#,^*p < 0.0332, ^##,^**p < 0.0021, ^###,^***p < 0.0002. Student’s two-tailed t-test was used for the analysis of differences between experimental groups (**F**–**M**). ^#,^*p < 0.05, ^##,^**p < 0.01, ^###,^***p < 0.001. Old-0% DSS, non-DSS-treated 18-week-old mice group; Old-Chronic and Old-Acute, 2% (w/v) chronic DSS-treated and 3% (w/v) acute DSS-treated 18-week-old mice group, respectively.

To further validate the causal association of DSS-induced colitis with hepatic steatosis, we performed the same experiments using older (18-week-old) mice as those presented above. For these experiments, two models (chronic and acute) of DSS-induced colitis were established using 18-week-old male mice. Chronic colitis was induced by 3 cyclic treatments with 2% DSS for 5 days followed by 5-days of water, and acute colitis was by 7 days of 3% DSS and 3 days of water. As shown in Fig. 6B–F, mice developed colitis having significantly decreased body weight and colon length with a significantly elevated DAI score in both chronic and acute colitis models. All the molecular and histological features of DSS-induced colitis exhibited in older mice (Fig. 6) were very similar to the case of younger mice (Fig. 1), which showed significant changes in DSS-treated groups, including decreased expression of genes related to intestinal integrity (Fig. 6G), elevated level of plasma LPS (Fig. 6H), upregulated colonic expression of pro-inflammatory cytokines (Fig. 6I), and increased transmural inflammation and crypt destruction with higher inflammation score (Fig. 6J), compared to non-DSS controls. In these chronic and acute DSS-induced colitis older mice, despite the significant reduction in body weight and the weights of adipose tissues, the liver weight was rather significantly increased in comparison with non-colitis control mice with commensurate increases in ectopic adiposity in the liver (Fig. 6K,L), which was the same pattern as that of younger mice (Fig. 2A–C). In addition, consistently with the results in the younger DSS-induced colitis mice, the older mice models also showed a more atherogenic lipid profile, characterized by significantly lower HDL-cholesterol and higher LDL-cholesterol levels than that of their non-colitis controls (Fig. 6M). Taken together with the data obtained from the experiments using younger mice, all findings observed in older mice evidently demonstrated that DSS-induced colitis caused the development of hepatic steatosis and dyslipidemia in mice.

### DSS-induced colitis older mice also have a similar molecular pathological phenotype to that found in their younger counterparts

**Figure 7:**
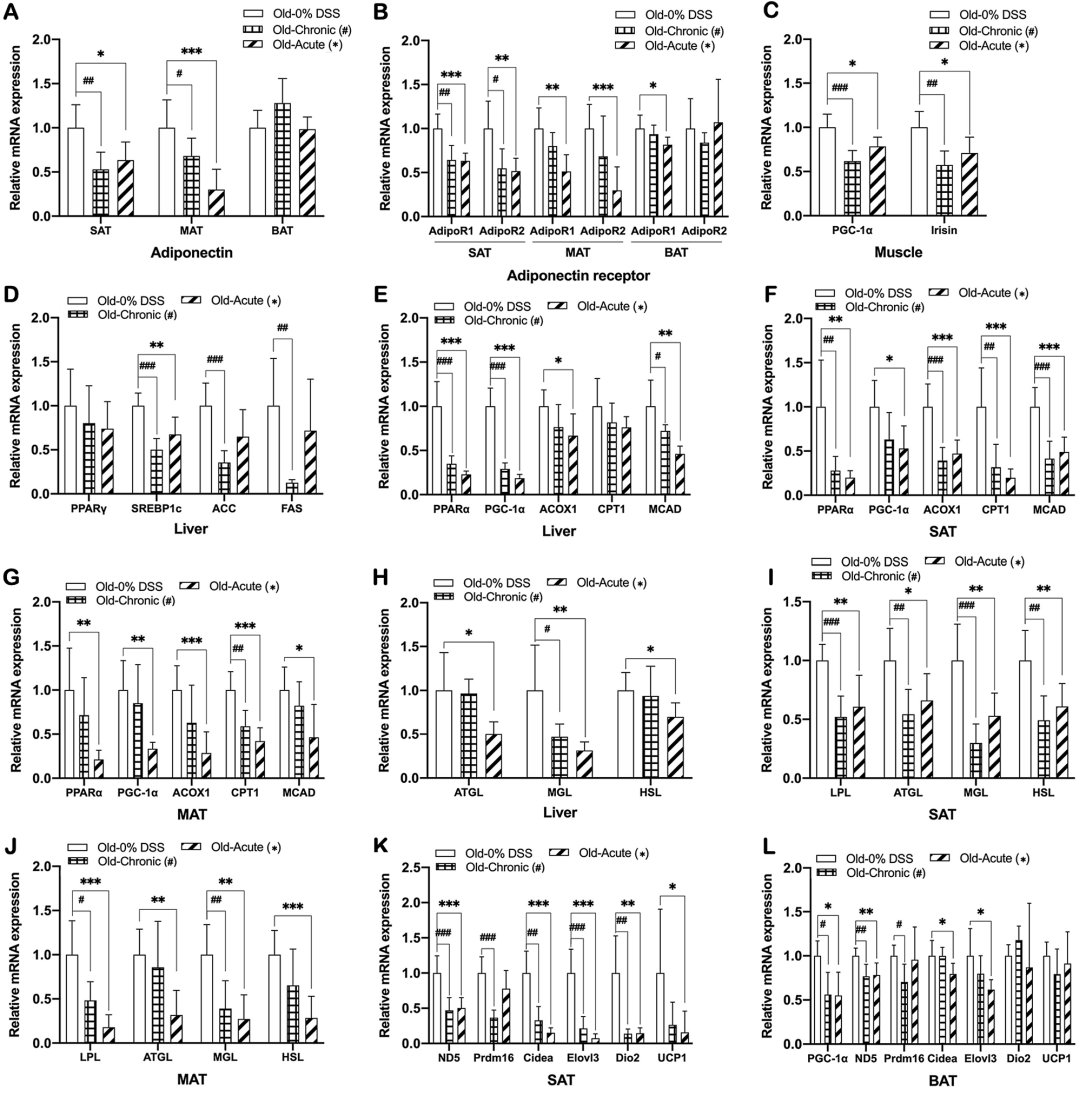
DSS-induced colitis older mice also have a similar molecular pathological phenotype to that found in their younger counterparts. mRNA expression of (**A**,**B**) adiponectin and adiponectin receptor in SAT, MAT, and BAT, (**C**) PGC-1α and irisin in the muscle, (**D**) lipogenic genes in the liver, (**E**–**G**) fatty acid oxidation genes in the liver, SAT, and MAT, (**H**–**J**) lipolytic genes in the liver, SAT, and MAT, (**K**) browning genes in SAT, (**L**) thermogenic genes in BAT of DSS-induced colitis and non-colitis 18-week-old mice. Gene expression levels are normalized to Arbp expression. Data present mean ± SD for 6–7 mice in each group. Student’s two-tailed t-test was used for the analysis of differences between experimental groups. ^#,^*p < 0.05, ^##,^**p < 0.01, ^###,^***p < 0.001.

To confirm whether the DSS-induced colitis mice at an older age developed similar features of metabolic dysfunctions with hepatic steatosis and dyslipidemia that was evident in their younger counterparts, we examined changes in expression of same metabolic regulators as those measured in younger mice. Both chronic and acute DSS-induced colitis older mice also showed changes in metabolic parameters in the similar pattern to that of younger mice, i.e., significant reductions in expression of adiponectin and adiponectin receptor (in SAT and MAT), PGC-1α and irisin (in muscle), lipogenic genes (in the liver), genes related to lipid oxidation and lipolysis (in the liver, SAT, and MAT), and genes involved in adipocyte browning (in SAT), compared to non-colitis controls (Fig. 7A–K). Just one difference was that thermogenic gene expression in BAT of older mice was not altered by colitis induction (Fig. 7L), while it was significantly reduced in younger mice (Fig. 5C). Together, these data indicated that molecular mechanisms underlying the development of DSS-induced colitis-associated metabolic dysfunctions in older mice were also very similar to those in younger counterparts and, regardless of whether the mice were at young or old ages, DSS-induced colitis was associated with the development of hepatic steatosis and dyslipidemia.

## Discussion

Although high NAFLD prevalence among patients with IBD has been frequently reported^14^, NAFLD pathogenic IBD-related factors and their related mechanisms, particularly at the molecular level, are still poorly understood. Our study aimed to explore the molecular mechanisms driving the development of NAFLD under IBD conditions using DSS-induced colitis model mice. To confirm the colitis-associated pathogenic effect, experiments with increasing concentrations of DSS ranging from 1 to 3% were performed. Additionally, in order to determine whether the relationship between the two diseases is common regardless of the patient's age, the results obtained from replicate experiments using mice at a younger (8-week-old) and an older (18-week-old) age were compared. Treatment of acute or chronic DSS induced the development of colitis in all younger and older mice tested, which was evidenced by increased weight loss, elevated DAI and histological inflammation score, and colon length shortening (Figs. 1, 6). We found that induction of chronic, but not acute, colitis with DSS in younger mice caused hepatic steatosis and dyslipidemia, which was consistently observed also in both chronic and acute DSS-induced colitis mice at older age (Figs. 2A–C, 4B, 6K–M).

Given the fact that endotoxemia is one of the common features of IBD and metabolic disorders, we hypothesized that an impaired intestinal barrier function caused by DSS treatment triggers LPS-induced systemic inflammation that could lead to disturbed energy homeostasis. Several studies have shown that in the pathogenesis of DSS-induced colitis, the intestinal epithelial barrier dysfunction is associated with the disruption of tight junction proteins^45,46^, as well as mucus layer proteins^47^. Mice with DSS-induced colitis used in this study consistently showed decreased expression of ZO-1, occludin, MUC2, and MUC13 (Figs. 1F–H, 6G), and elevated level of plasma LPS (Figs. 1I, 6H), indicating an impairment of intestinal barrier functions and thereby increased intestinal permeability. It has been demonstrated that the loss of intestinal barrier integrity triggers a cascade of events resulting in systemic inflammation, eventually leading to both intestinal and systemic diseases including IBD and metabolic disorders^5,48^. Abnormalities in intestinal immune responses associated with impaired barrier function include an imbalance between the pro- and anti-inflammatory cytokines, which is known to play an important role in the pathogenesis of IBD^49–51^. In this study, DSS-induced colitis mice showed substantially higher levels of pro-inflammatory cytokines (Figs. 1J, 6I), a lower level of anti-inflammatory M2 macrophage (Fig. 1L), and higher levels of immune cell infiltration when compared to non-colitis controls (Figs. 1M, 6J), indicating an impairment of the intestinal immune system caused by DSS-induced barrier dysfunction. Our findings also show that DSS-induced colitis caused upregulation of pro-inflammatory cytokines and ER stress-related proteins in peripheral tissues such as the liver and adipose tissue (Fig. 2D–F), implying that DSS treatment disrupted intestinal barrier integrity and increased circulating LPS, thus leading to systemic inflammation. There are several shreds of evidence that fatty liver diseases are causally associated with impaired intestinal permeability^52,53^. Increased LPS in systemic circulation as a consequence of intestinal barrier dysfunction is causally linked to the pathogenesis of multiple diseases, including NAFLD^54^. In the liver, LPS binds to their receptors on the Kupffer cells and stimulates the production of pro-inflammatory cytokines, leading to the development of hepatic inflammation and necrosis^55–57^. These reports are consistent with our observations that there were significant increases in hepatic pro-inflammatory cytokines and ER stress markers (Fig. 2D–F) as well as plasma AST and ALT (Fig. 2G) in DSS-treated mice, which demonstrates that DSS-induced loss of intestinal barrier integrity with increased permeability results in LPS-related endotoxemia, causing chronic hepatic inflammation and damage.

To explore the molecular mechanism underlying the development of hepatic steatosis and dyslipidemia in DSS-induced colitis mice, considering the critical role of crosstalk between intestine and other metabolic organs in energy homeostasis, we next hypothesized that DSS-induced barrier dysfunction might cause a disturbance of inter-organ metabolic homeostasis, thereby resulting in impaired lipid metabolism in the liver and adipose tissues. Several lines of evidence have demonstrated that hepatocellular fat accumulation in NAFLD is linked to an imbalance in fatty acid uptake, de novo lipogenesis, fatty acid oxidation and lipid export^58^, which is caused by dysregulation of mediators for inter-organ metabolic communication such as FGF21, adiponectin, and irisin, as well as their key regulator SIRT1^20–22,28^. SIRT1 plays essential roles in controlling lipid and cholesterol homeostasis. For instance, in the liver, it promotes fatty acid oxidation through PPARα/PGC-1α-mediated pathway^30^ and enhances RCT by up-regulating the LXR target gene ABCA1, resulting in increased HDL biogenesis^31^. It also drives WAT browning through PPARγ deacetylation^59^ and promotes BAT thermogenesis through up-regulation of PGC-1α-mediated mitochondrial genes^60^. Furthermore, SIRT1 plays a protective role against hepatic inflammation by blocking NF-κB-mediated induction of pro-inflammatory cytokines^61^, while in chronic inflammatory conditions SIRT1 is dysregulated^62^. However, although SIRT1 has been intensively studied as a master regulator in the pathogenesis of chronic inflammation-related metabolic complications, it has not been known whether its dysfunction is associated with DSS-induced colitis. Our findings in the present study show that DSS-induced colitis causes a disturbance in hepatic lipid metabolism through the disruption of SIRT1-mediated metabolic processes including fatty acid oxidation, RCT, WAT browning, and BAT thermogenesis. We observed that SIRT1 expression was significantly downregulated in the liver, SAT, and BAT of DSS-induced colitis mice (Fig. 2H), which was accompanied by suppressed expression of fatty acid oxidation genes in the liver, SAT, and MAT (Figs. 3C–E, 7E–G). Colitis-associated downregulation of genes involved in RCT (Fig. 4E), SAT browning (Figs. 5A,B, 7K), and BAT thermogenesis (Figs. 5C, 7L) was also evident in acute or chronic DSS-induced colitis mice. These data collectively suggest a chronic inflammatory condition caused by DSS-induced intestinal barrier dysfunction reduces hepatic SIRT1 expression and consequently suppresses SIRT1-mediated metabolic processes, which at least in part contributes to the development of hepatic steatosis and hypo-HDL cholesterolemia. Accumulated evidence shows that adiponectin downregulation is also a key feature of chronic inflammation in metabolic disorders^63^. Under these inflammatory conditions, cellular NAD^+^ levels are decreased in metabolic organs resulting in reduced activity of the NAD^+^-dependent deacetylase SIRT1, which is reversed by adiponectin via AMPK activation and subsequent induction of SIRT1/PGC-1α mediated mitochondrial functions including fatty acid oxidation, WAT browning, and BAT thermogenesis^33,64^. Thus, the deactivation of adiponectin-AMPK-SIRT1 pathway caused by chronic inflammation might be one potential mechanism for hepatic steatosis associated with DSS-induced colitis. Our data showing a downregulation of adiponectin, AdipoR1, and AdipoR2 in the liver and adipose tissues and a reduction in AMPK activation in the liver of DSS-induced colitis mice (Fig. 2I–L), together with reduced expression of SIRT1 (Fig. 2H), suggest that colitis might cause a disturbance in adiponectin-AMPK-SIRT1 mediated metabolic regulation. LXRs are also key metabolic regulators of fatty acid and cholesterol homeostasis^65^, and after being deacetylated by SIRT1, promote the expression of genes involved in RCT^66^ as well as hepatic lipogenesis^67^. The results of this study that showed a reduction of LXRα expression accompanied by downregulated gene expressions related to both RCT and lipogenesis in the liver of DSS-induced colitis mice (Figs. 3A, 4E), suggest that DSS-induced colitis also perturbs LXRα-mediated RCT and hepatic lipogenesis.

However, these findings remained a question why, despite a suppressed lipogenesis, hepatic fat accumulation is increased as shown in Fig. 2A–C. Thus, we directed our focus to the comprehensive analysis of lipid metabolic pathways including lipogenesis, lipolysis, fatty acid oxidation, and fatty acid uptake. Data obtained in this study reveal that DSS-induced colitis is associated with decreased expression of not only the genes responsible for hepatic lipogenesis (Figs. 3A,B, 7D) but also the genes of lipid oxidation and lipolysis in the liver and adipose tissues (Figs. 3C–I, 7E–J). DSS-induced colitis-associated suppression of fatty acid uptake and lipid export was also evident from decreased expression of genes in those metabolic pathways in the liver (Fig. 3J–L). Collectively, these results suggest a possible explanation that DSS-induced colitis causes perturbations in overall lipid metabolism in the liver and adipose tissues, which implies that abnormal fat accumulation generated by impaired fatty acid oxidation, lipolysis, and lipid export might possibly override fat reduction due to impaired fatty acid uptake and lipogenesis, consequently developing a state of hepatic steatosis.

**Figure 8:**
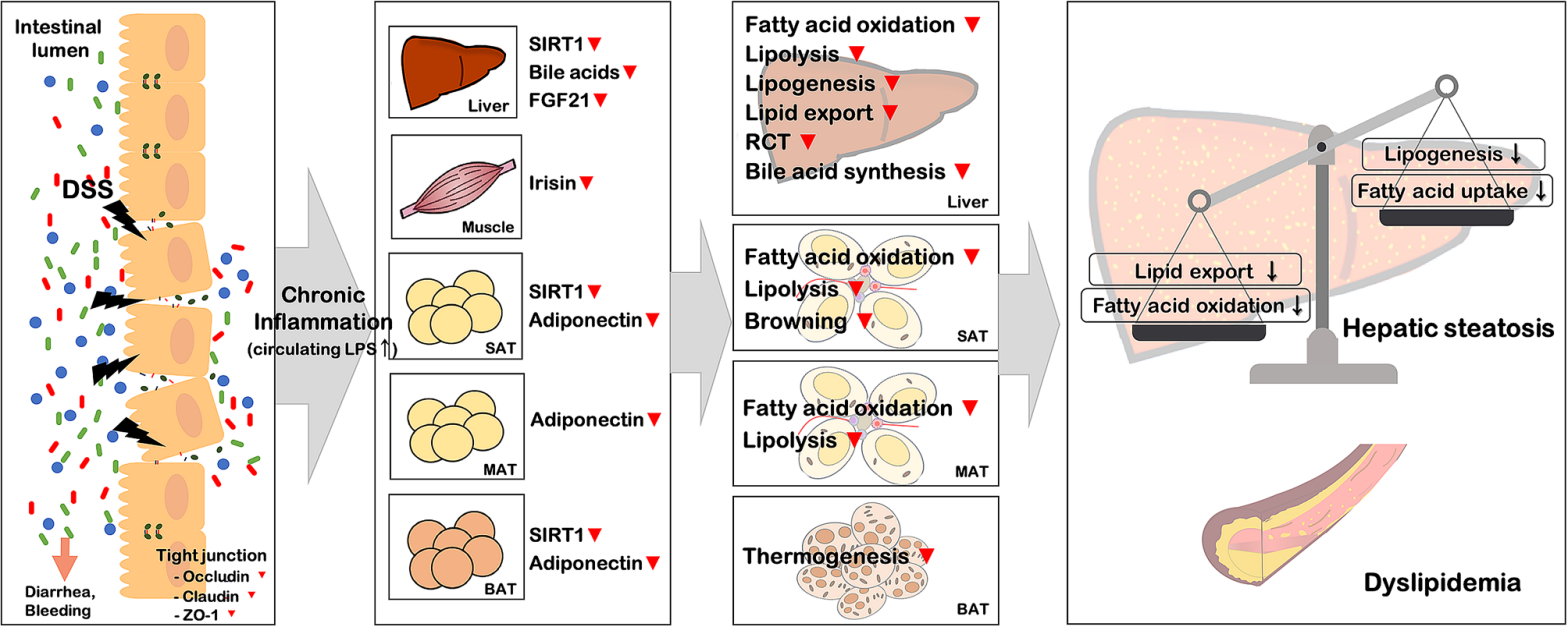
A potential molecular mechanism for how DSS-induced colitis is linked to metabolic-dysfunction phenotypes, i.e. hepatosteatosis and dyslipidemia.

Browning of WAT and thermogenesis in BAT, being considered as major contributors to energy expenditure, also impact systemic lipid homeostasis. Multiple secreted factors including FGF21, irisin, adiponectin, and bile acids have been identified to activate these two metabolic processes. FGF21 and irisin promote PGC-1α-mediated mitochondrial biogenesis and UCP1 expression to enhance browning program in WAT, especially SAT, and thermogenic activity of BAT, eventually improving energy metabolic processes including hepatic lipid metabolism^36,68^. Bile acids are also recognized as key metabolic regulators with their functions to increase SAT browning and BAT thermogenesis by promoting thyroid hormone activation via TGR5-cAMP-Dio2 signaling^69^. The results of this study demonstrate that DSS-induced colitis is associated with suppressed gene expressions related to SAT browning and BAT thermogenesis (Figs. 5A–C, 7K,L), and their master regulators, PPARα and PGC-1α (Figs. 3D, 5C, 7F,L) in both SAT and BAT. These changes were also accompanied by significant decreases in expression of irisin in skeletal muscle (Figs. 2O,P, 7C), FGF21 (Fig. 2M,N) and bile acid synthetic genes in the liver (Fig. 4F), SIRT1 in the liver, SAT, and BAT (Fig. 2H), TGR5 in most metabolic tissues (Fig. 4G), and GLP-1 in the intestine (Fig. 4H). These findings suggest that DSS-induced colitis may cause impairment of SAT browning and BAT thermogenesis through suppressing the expression of key regulators of energy balance, including irisin, FGF21, bile acids, and SIRT1. Taken together, our findings in this study suggest that DSS-induced colitis might have perturbing effects on overall metabolic processes involved in energy homeostasis including fatty acid oxidation, lipolysis, fatty acid uptake, lipogenesis, cholesterol synthesis, RCT, bile acid synthesis, WAT browning, and BAT thermogenesis in mice, which eventually display hepatic steatosis- and dyslipidemia-like phenotypes. Most of these metabolic pathways being impaired by colitis are SIRT1/PGC-1α- or LXRα-mediated ones. Considering the high prevalence of NAFLD in patients with IBD, the discovery of molecular mechanism for the association of intestinal inflammatory conditions with fatty liver-related metabolic disorders such as steatosis and dyslipidemia is important. In the present study, our results suggest a possible mechanism that may underlie NAFLD phenotypes in patients with IBD, which is related to main energy metabolic processes regulated via SIRT1/PGC-1α- and LXRα-mediated pathways by key metabolic regulators such as adiponectin, FGF21, and irisin. Here, as shown in Fig. 8, we propose a model that explains how DSS-induced colitis is linked to metabolic-dysfunction phenotypes, i.e. hepatosteatosis and dyslipidemia. However, it is also important to note that, although the changes in mRNA expression levels for many examined genes were detected in this study, not all of those genes were confirmed to show the same trend also in protein expression levels. Given the fact that mRNA levels do not necessarily reflect protein expression or the functional activity of the corresponding protein, our study has a limitation that the generated model is not an unquestionable interpretation, and further studies are needed to confirm the accuracy of the model as the exact link mechanism.

In summary, DSS-induced colitis mice displayed NAFLD phenotype such as hepatosteatosis and dyslipidemia, which was age-independent. This DSS-induced colitis-associated lipid metabolic dysfunction was most likely due to overall disruption of metabolic functions such as fatty acid oxidation, lipolysis, RCT, bile acid synthesis, and WAT browning and BAT thermogenesis, most of which are mediated via SIRT1/PGC-1α and LXRα. The present study provides a potential molecular mechanism underlying the development of NAFLD as a frequent comorbidity in IBD patients, which could provide a key to understanding what common factors the two diseases share in their pathogenesis and discovering critical therapeutic targets for their treatment.

## Methods

### Mice

Six-week-old C57BL/6L male mice purchased from Central Lab. Animal, Inc. (Seoul, Korea) were housed under standard laboratory conditions (22 ± 1 °C, 12:12-h light/dark cycle). All mice were given ad libitum access to a normal chow diet (SAFE R40-10, Safe Complete Care Competence, Augy, France), and water throughout the study. All animal experiments were performed in accordance with protocols approved by the Animal Care and Use Committee of the Handong Global University (Approval No. HGUIACUC20191008-020). This study was carried out in compliance with the ARRIVE guidelines.

### Chronic DSS-induced colitis mouse model

Colitis was induced by oral administration of DSS (MW 36–50 kDa, MP Biomedicals, Santa Ana, CA). After acclimation, 8-week-old mice were randomly assigned to 4 groups (n = 8 per group): 0 (negative control), 1, 2, and 3% (w/v) DSS-treated group. For the chronic DSS-induced colitis, mice were treated with 3 cycles of DSS for 7 days and 7 days of drinking water between each cycle. Body weight, stool softness, and blood in the rectum or stool were recorded daily during DSS and recovery cycles. After the 3rd cycle of DSS treatment, mice were sacrificed and tissue samples were harvested as previously described^70^. When colitis was induced in 18-week-old male mice, mice were divided into 3 groups: non-DSS-treated control (n = 6), chronic DSS-induced colitis (2% DSS, n = 7), and acute DSS-induced colitis (3% DSS, n = 7) group. For the chronic DSS-induced colitis, mice were treated with 3 cycles of DSS for 5 days and 5 days of drinking water between each cycle. For the acute DSS-induced colitis, mice were treated with 7 days of DSS and 3 days of drinking water right after the treatment (Fig. 6A).

### Assessment of colitis severity

During the DSS treatment cycles, a disease activity index (DAI) score was assessed to evaluate the progression of colitis. The DAI was a composite score determined by factors such as relative body weight loss, stool softness, and blood in the rectum or stool^71^. Body weight loss was scored as follows: score 0, no weight loss compared to initial weight; 1, weight loss within 1–5%; 2, weight loss within 5–10%; 3, weight loss within 10–20%; 4, greater than 20% weight loss. Stool consistency was determined as follows: score 0, normal (solid pellet); 1, soft but in pellet shape; 2, loose stool but with some solidity; 3, loose stool with signs of liquid consistency; 4, watery diarrhea. Rectal bleeding was scored as follows: score 0, no sign of blood; score 1, no bleeding; 2, slight bleeding; 3, bloody diarrhea; 4, gross bleeding.

To analyze the severity of colonic inflammation, Swiss rolls were made from colon tissues and hematoxylin and eosin (H&E) stained for the histomorphological evaluation. Fresh colon tissues were washed with cold PBS, cut longitudinally into 3 parts, from one of which Swiss roll was prepared by fixing in PBS-buffered 10% formalin, embedding them in paraffin, and then sectioning into 5-μm thick slices for H&E staining. The colitis severity was measured in a blinded manner by two professional pathologists. Briefly, the degree of colon inflammation was scored using light microscopy to look for signs of inflammation, cell infiltration, fibrosis, and epithelial and mucosal damage. The observers scored the tissue morphology as follows: the presence of inflammatory cells such as polymorphonuclear cells, lymphocytes, plasma cells, macrophages, and giant cells per high-power (400×) field (phf) was scored as 0, normal tissue; 1, rare (1–5/phf); 2, slight increase (5–10/phf); 3, more obvious increase; 4, significantly higher increase (packed), the extent of the leukocyte infiltration was scored as 0, rare (1–5/phf); 1, significant increase in lamina propria (5–10/phf); 2, confluence in the submucosal part; 3, transmural infiltration, the severity of fibrosis was scored as 0, normal; 1, mild; 2, moderate; 3, severe; 4, very severe, the epithelial damage was scored as 0, normal tissue; 1, loss of the basal one-third of the crypt; 2, loss of the basal two-third of the crypt; 3, entire crypt loss; 4, focal erosion; 5, confluent erosion, and the mucosal damage was scored as 0, normal tissue; 1, 1 or 2 foci of ulcerations; 2, 3 or 4 foci of ulcerations; 3, confluent or extensive ulceration^72^.

### Plasma analyses

For collection of blood plasma, fresh blood obtained via cardiac puncture was collected in a BD Microtainer PST tube (BD Scientific, Franklin Lakes, NJ) and subsequently centrifuged at 15,000*g* for 5 min at 18 °C, and the separated plasma was frozen at − 70 °C until analysis. Plasma levels of triglyceride (TG), total cholesterol, high-density lipoprotein (HDL) cholesterol, low-density lipoprotein (LDL) cholesterol, AST, and ALT were measured using an automated analyzer, Mindray BS-390 (Mindray Bio-Medical Electronics Co., Shenzhen, China). Plasma lipopolysaccharide (LPS) level was measured using ToxinSensor Chromogenic LAL Endotoxin Assay Kit (GenScript, Piscataway, NJ).

### Real-time RT-PCR

Total RNA was extracted using NucleoZOL (Macherey‐Nagel, Düren, Germany) and complementary DNA was synthesized by reverse transcription of 2 μg of extracted RNA using the GoScript reverse transcription system (Promega, Madison, WI) according to the manufacturer’s instructions. Quantitative real-time PCR was performed using GoTaq qPCR Master Mix (Promega) on a StepOnePlus Real-Time PCR system (Applied Biosystems, Foster City, CA). Quantification of gene transcripts for acidic ribosomal phosphoprotein (Arbp), glyceraldehyde-3-phosphate dehydrogenase (GAPDH), ZO-1, occludin, MUC2, MUC13, interleukin 17 (IL-17), tumor necrosis factor α (TNFα), IL-1β, interferon γ (IFNγ), IL-4, IL-6, IL-22, IL-23, F4/80, cluster of differentiation 4 (CD4), CD8, forkhead box P3 (FOXP3), CD19, integrin subunit beta 7 (Itgb7), CD163, CD206, monocyte chemoattractant protein-1 (MCP-1), immunoglobulin heavy chain-binding protein (BiP/GRP78), CCAAT-enhancer-binding protein homologous protein (CHOP), adiponectin, adiponectin receptor 1 (AdipoR1), AdipoR2, peroxisome proliferator-activated receptor α (PPARα), PGC-1α, acyl-CoA oxidase 1 (ACOX1), carnitine palmitoyltransferase 1 (CPT1), medium-chain acyl-coenzyme A dehydrogenase (MCAD), lipoprotein lipase (LPL), adipose triglyceride lipase (ATGL), monoacylglycerol lipase (MGL), hormone-sensitive lipase (HSL), CD36, low density lipoprotein receptor (LDLR), PPARγ, sterol-regulatory element binding protein 1c (SREBP1c), acetyl-CoA carboxylase (ACC), fatty acid synthase (FAS), stearoyl-CoA desaturase 1 (SCD1), diacylglycerol acyltransferase 1 (DGAT1), 2 (DGAT2), glycerol-3-phosphate acyltransferase (GPAT), SREBP2, HMG-CoA reductase (HMGCR), HMG-CoA synthase (HMGCS), scavenger receptor class B type 1 (SR-B1), ATP-binding cassette subfamily G member 5 (ABCG5), ABCG8, ABCA1, apolipoprotein A1 (ApoA1), ApoB, microsomal triglyceride transfer protein (MTP), liver X receptor α (LXRα), cholesterol 7 alpha-hydroxylase (CYP7A1), CYP7B1, CYP27A1, CYP8B1, bile salt export pump (BSEP), SIRT1, TGR5, FGF21, irisin, NADH-ubiquinone oxidoreductase chain 5 (ND5), Prdm16, Cidea, Elovl3, Dio2, uncoupling protein 1 (UCP1), and GLP-1 was performed using gene-specific forward and reverse primers. Primer sequences are available in Supplementary Table S2. The relative expression levels of each gene were calculated using the ΔΔ Ct method and normalized to the expression of Arbp or GAPDH.

### Histological analysis of the liver tissue

For histological examination, the liver tissue specimens were fixed in 10% buffered formalin, embedded in paraffin, cut at 5-μm thicknesses, and stained with hematoxylin and eosin (H&E). Images were obtained under a Carl Zeiss light microscope (Carl Zeiss Microscopy GmbH, Göttingen, Germany) at a magnification of 400×.

### Hepatic lipid analyses

Triglycerides (TG) levels in the liver were quantified by colorimetric assay using the TG Assay kit (Asan Pharmaceutical, Seoul, Korea) according to the manufacturer’s protocol. Briefly, liver tissue samples were homogenized in 5% (w/v) aqueous solution of NP-40, and samples were slowly heated to 100 °C for 5 min and cooled to room temperature, and heating–cooling cycle was repeated two times more, and then the lysates were centrifuged at 15,000×*g* for 2 min. The supernatant was collected, diluted five times with ddH_2_O, and mixed with TG assay buffer (TG Assay kit, Asan Pharmaceutical) to measure absorbance at 550 nm. Total cholesterol levels in the liver were quantified by colorimetric assay using the Total Cholesterol Assay kit (Asan Pharmaceutical). Liver tissue samples were homogenized in chloroform/isopropanol/NP-40 (7:11:0.1) solution, and the lysates were centrifuged at 15,000×*g* for 10 min, incubated for 2 h at 50 °C, and then dried at vacuum incubator for 30 min. Dried samples were mixed with total cholesterol assay buffer (Asan Pharmaceutical) to measure absorbance at 500 nm.

### Western blot analysis

Western blotting was performed as described previously^70^. Antibodies against AMPK, BiP, CD36, CHOP, FAS, FGF21, HSL, occludin, phospho-AMPK (Thr172), phospho-HSL (Ser563), SCD1, UCP1, β-actin, and GAPDH were used as primary antibodies, followed by anti-rabbit IgG-HRP conjugated secondary antibody. Immunoblots were visualized by ECL, and densitometric analyses were done using ImageJ software. Detailed information on primary and secondary antibodies used in this study are listed in Supplementary Table S3.

### Statistical analyses

All data were presented as means ± SD for 5–8 mice per each group. Statistical significance between groups was analyzed using GraphPad Prism software version 8.0 (GraphPad, San Diego, CA). For the analysis of data with measures of body weight and DAI, a two-way analysis of variance (ANOVA) with Dunnett’s multiple comparison test was used. *P* values < 0.0332 were considered as statistically significant. In the cases of plasma analyses, real-time PCR, Western blotting experiments, and hepatic lipid analyses, the student’s two-tailed t-test was used to determine statistical significance between experimental groups. *P* values < 0.05 were considered as statistically significant.

## Supplementary Information

Supplementary Information.

## Data Availability

All relevant data are within the paper and its Supplementary Information files.
