# Supplementary material for: DSS-induced colitis is associated with adipose tissue dysfunction and disrupted hepatic lipid metabolism leading to hepatosteatosis and dyslipidemia in mice
Source: Sci Rep. 2021 Mar 5;11:5283. doi: 10.1038/s41598-021-84761-1 (PMC7935975; doi:10.1038/s41598-021-84761-1)
Supplement: Supplementary file 1 — Supplementary Information. [file 41598_2021_84761_MOESM1_ESM.docx]

**DSS-induced colitis is associated with adipose tissue dysfunction and disrupted hepatic lipid metabolism leading to hepatosteatosis and dyslipidemia in mice**

Jeonghyeon Kwon, Chungho Lee, Sungbaek Heo, Bobae Kim, and Chang-Kee Hyun*

School of Life Science, Handong Global University, Pohang, Gyungbuk 37554, South Korea

* Corresponding author

E-mail: ckhyun@handong.edu (C.-K. Hyun)

Supplementary Figure S1. Food and water intake during the 3 cycles of DSS treatment. (A and B) Changes in food and water intake during 3 cycles of DSS treatment, respectively (n=5-8). Student’s two-tailed t-test was used for analysis of differences between experimental groups. ^•, +, *^*p* < 0.05, ^••, ++, **^*p* < 0.01, ^•••, +++, ***^*p* < 0.001.


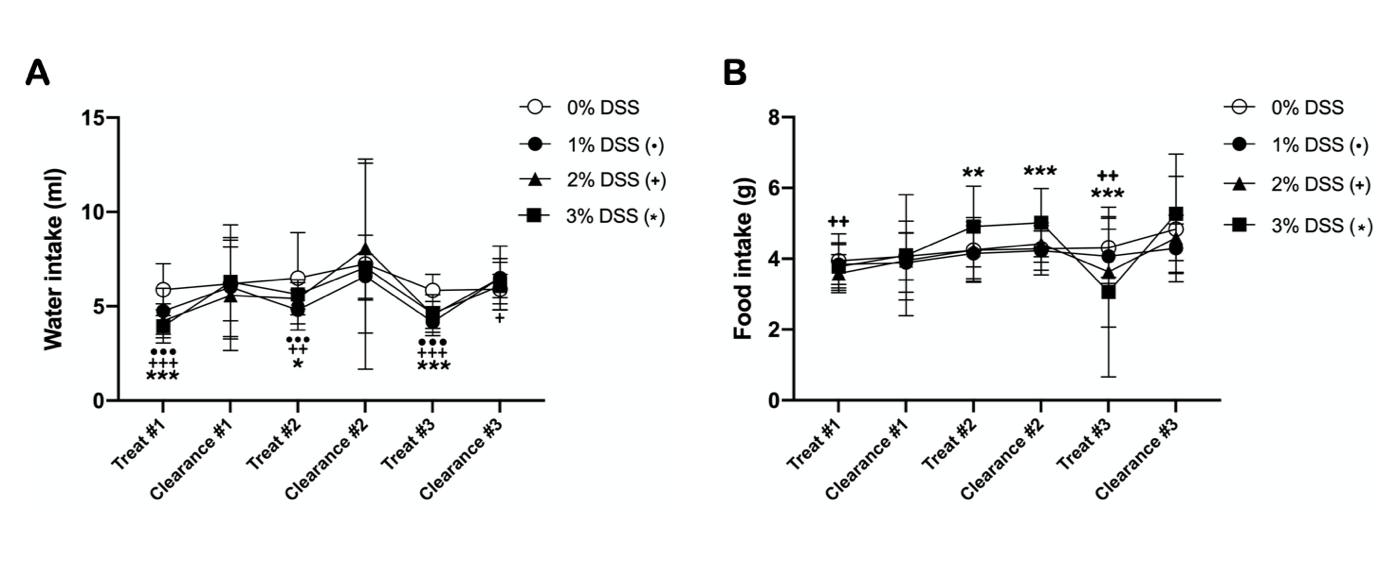


Supplementary Figure S2. Raw images of Figure 1F.


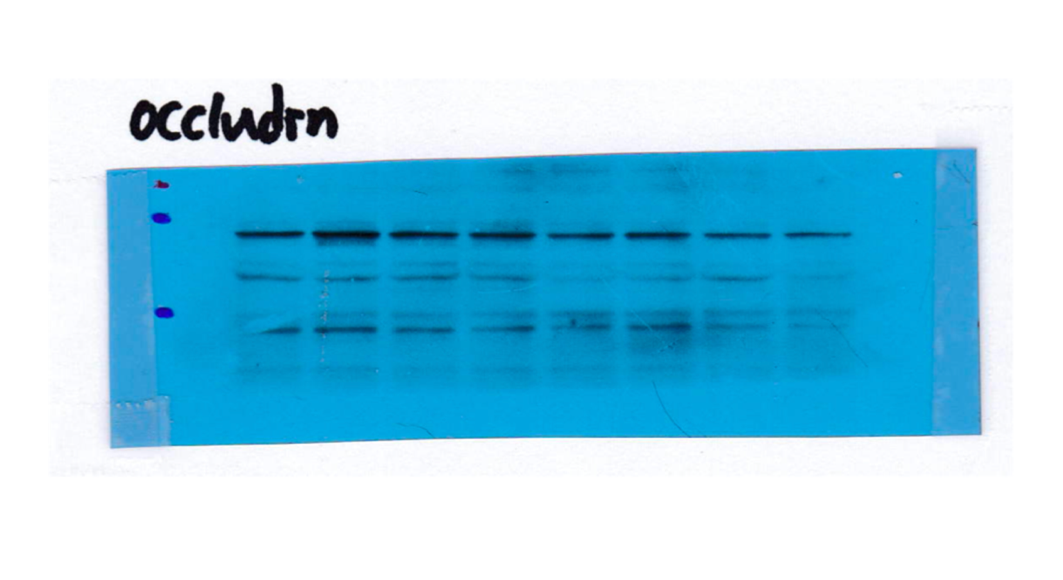
**
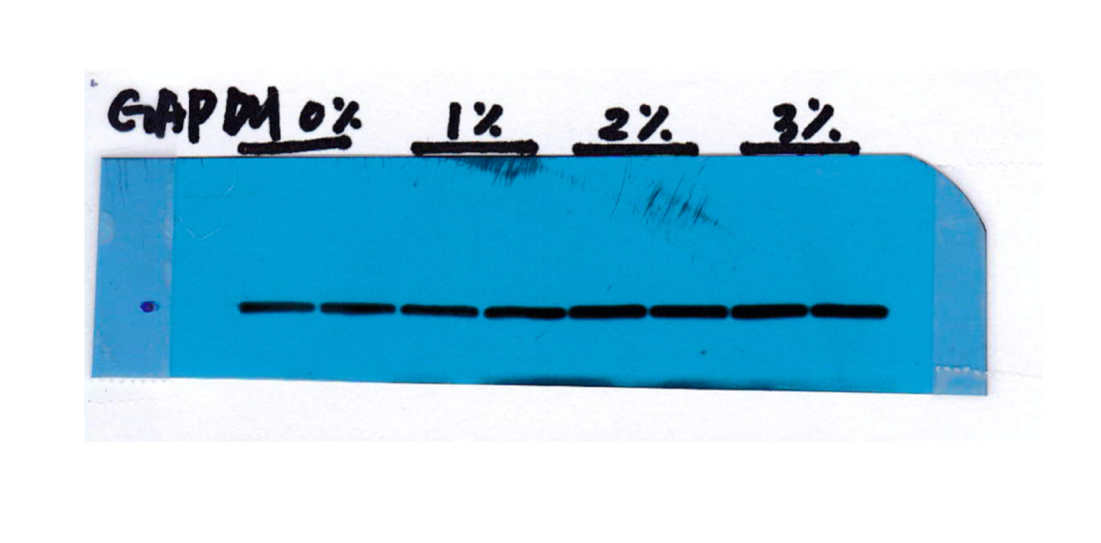
**

Supplementary Figure S3. Raw images of Figure 2F.

Liver


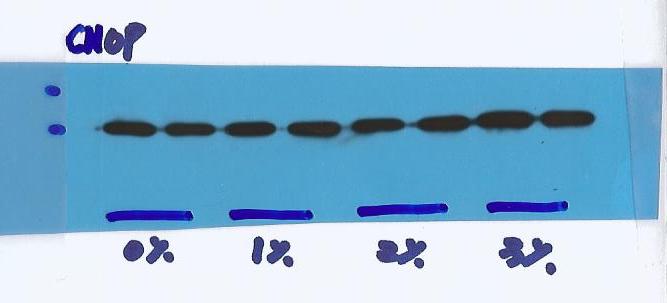

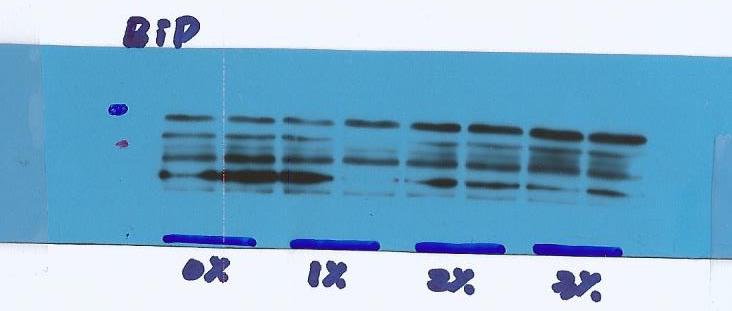


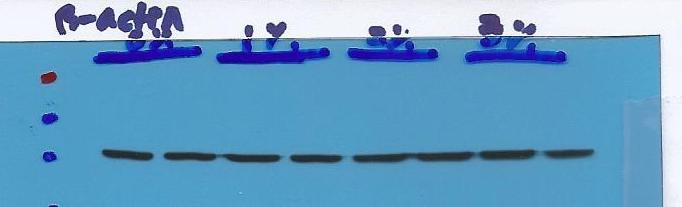


SAT


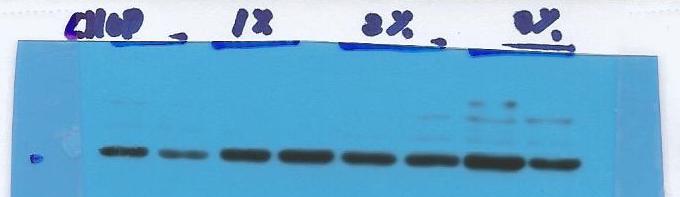


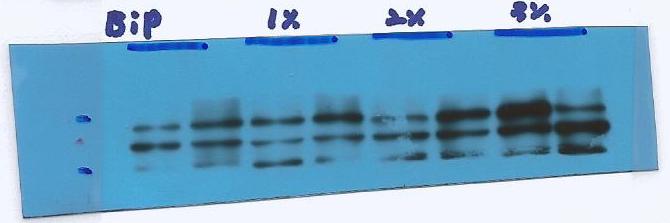


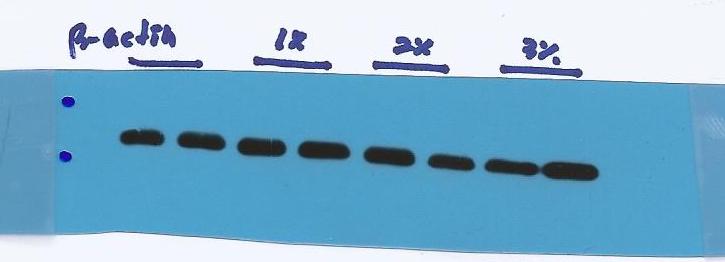


Supplementary Figure S4. Raw images of Figure 2J.


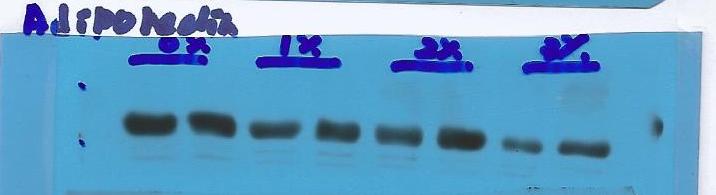


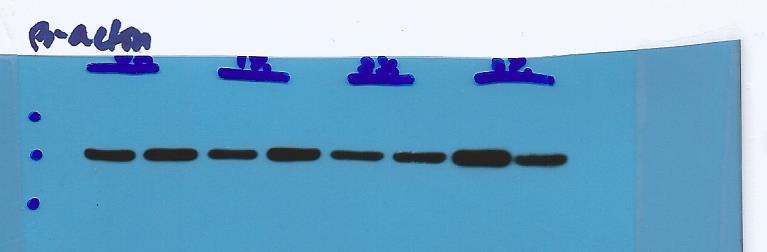


Supplementary Figure S5. Raw images of Figure 2L.


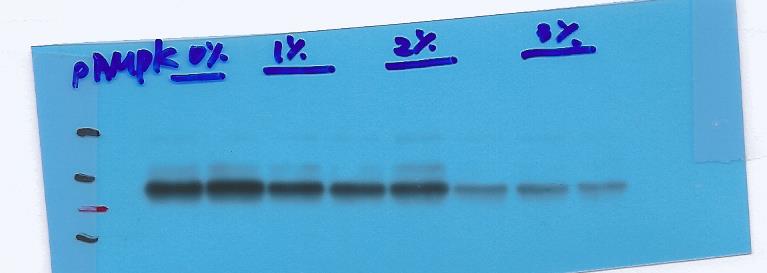


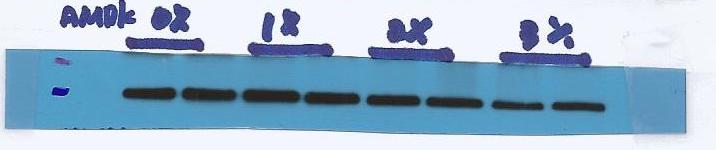


Supplementary Figure S6. Raw images of Figure 2N.


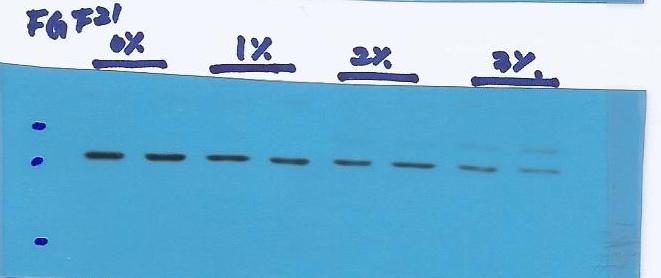


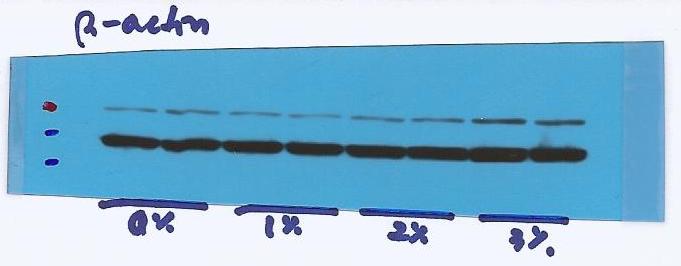


Supplementary Figure S7. Raw images of Figure 2P.


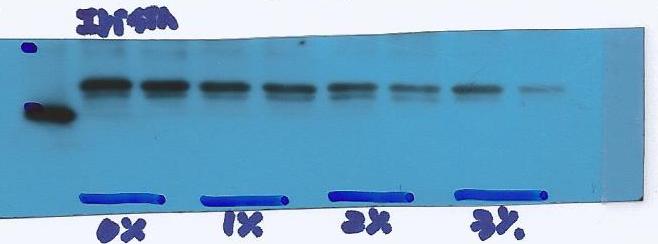


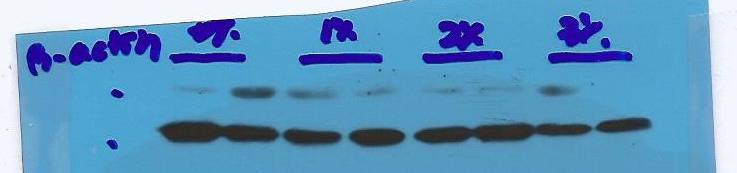


Supplementary Figure S8. Raw images of Figure 3B.


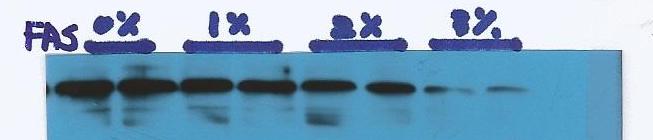


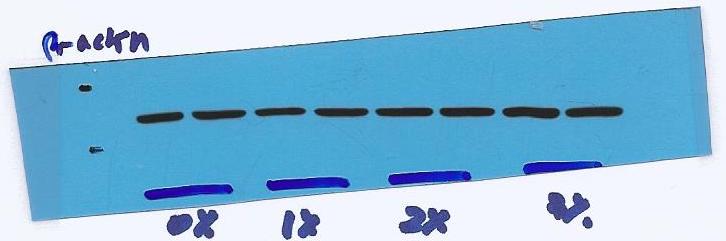


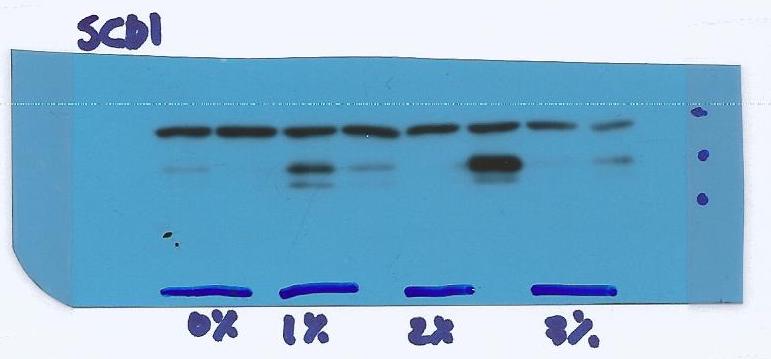


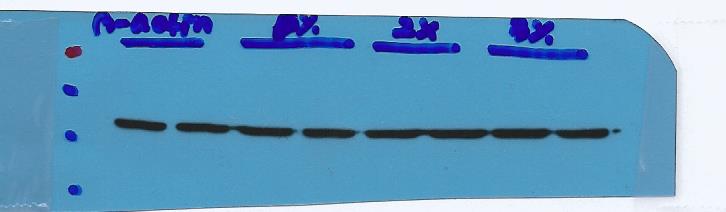


Supplementary Figure S10. Raw images of Figure 3G.


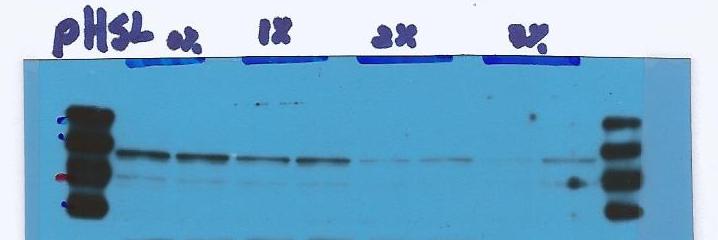


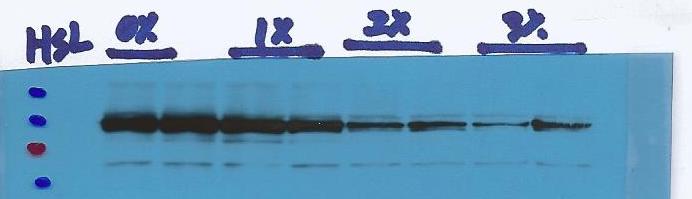


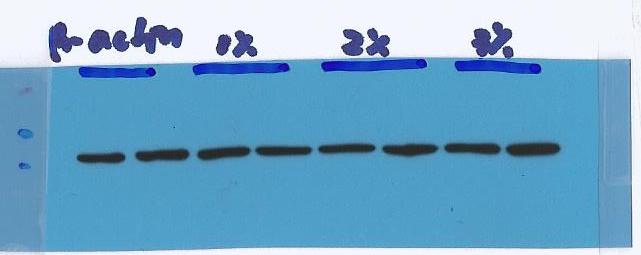


Supplementary Figure S11. Raw images of Figure 3L.


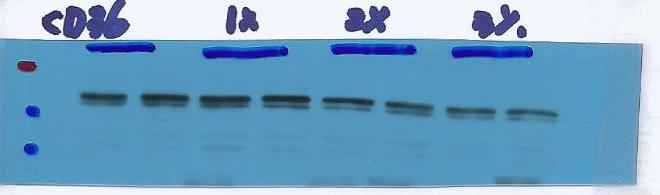


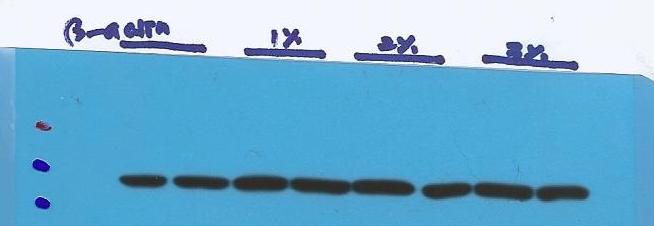


Supplementary Figure S12. Raw images of Figure 5B.


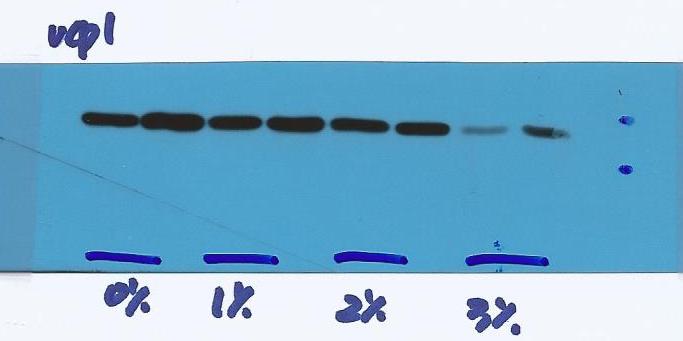


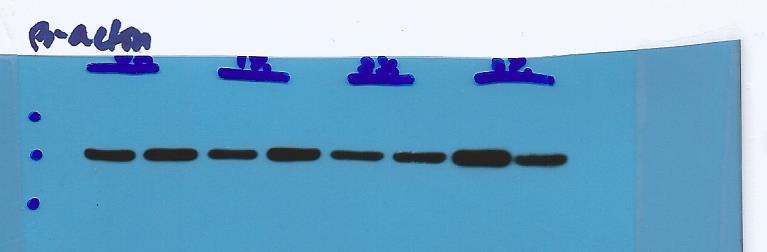


Supplementary Table S1. Composition of Diet

| **Diet (% w/w)** | **Normal Diet** |
| --- | --- |
| Protein | 15.2 |
| Carbohydrate | 61 |
| Cellulose | 4.1 |
| Lipid | 3.2 |
| Vitamin & Mineral mix | 4.4 |
| Moisture | 12.1 |
| Energy density (kcal/g) | 3.336 |

* Diets were not sterilized.

Supplementary Table S2. Primer sequences for real-time PCR

| **Gene** | | **Sequence** |
| --- | --- | --- |
| ABCA1 | Forward | GCGACCATGAAAGTGACACG |
|  | Reverse | CAGCACATAGGTCAGCTCGT |
| ABCG5 | Forward | CCTGCTGAGGCGAGTAACAA |
|  | Reverse | GGACGCGGAGAAGGTAGAAA |
| ABCG8 | Forward | GAAAAGAACCAGCGGGGAGA |
|  | Reverse | GCCTGGGATTTTGCCTACCT |
| ACC | Forward | TGACAGACTGATCGCAGAGAAAG |
|  | Reverse | TGGAGAGCCCCACACACA |
| ACOX1 | Forward | GTGCAGCTCAGAGTCTGTCCAA |
|  | Reverse | TACTGCTGCGTCTGAAAATCCA |
| Adiponectin | Forward | GCAGAGATGGCACTCCTGGA |
|  | Reverse | CCTTCAGCTCCTGTCATTCC |
| AdipoR1 | Forward | TGGCTGATAACGGGCCATC |
|  | Reverse | GGCGTGGCTTTGTTTGTCC |
| AdipoR2 | Forward | ACTCTGACAGGATTTGGGGTC |
|  | Reverse | GTGCCCTTTTCTGAGCCGTA |
| ApoA1 | Forward | GCACGTATGGCAGCAAGATG |
|  | Reverse | GGGACACATAGTCTCTGCCG |
| ApoB | Forward | AGGTACGAACTCAAGCTGGC |
|  | Reverse | GAGCAGAGATGATGCCCCTC |
| Arbp | Forward | TCACTGTGCCAGCTCAGAAC |
|  | Reverse | AATTTCAATGGTGCCTCTGG |
| ATGL | Forward | GACAGCTCCACCAACATCCA |
|  | Reverse | GCAAAGGGTTGGGTTGGTTC |
| BSEP | Forward | CTGCCAAGGATGCTAATGCA |
|  | Reverse | CGATGGCTACCCTTTGCTTCT |
| CD163 | Forward | GACTTGCTGAAATCCTCGGAAAA |
|  | Reverse | AGTGTGCCTCTGAATGACCC |
| CD19 | Forward | AACCACGTGACTCCCAAGTG |
|  | Reverse | TTCCTATCCCACACAAGGGC |
| CD206 | Forward | GTGGAGTGATGGAACCCCAG |
|  | Reverse | CTGTCCGCCCAGTATCCATC |
| CD36 | Forward | TCCTCTGACATTTGCAGGTCTATC |
|  | Reverse | AAAGGCATTGGCTGGAAGAA |
| CD4 | Forward | CCAGACAGTGTTCCTGGCTT |
|  | Reverse | TGCCTGGCGCTGTTGG |
| CD8 | Forward | TCCTGGCGGTGCCATTTTAC |
|  | Reverse | TCCTGGCGGTGCCATTTTAC |
| CHOP | Forward | AGTACTGGCTCCGTCTAACCC |
|  | Reverse | GACACCGTCTCCAAGGTGAAA |
| Cidea | Forward | CTAGCACCAAAGGCTGGTTC |
|  | Reverse | CACGCAGTTCCCACACACTC |
| CPT1 | Forward | TGAGTGGCGTCCTCTTTGG |
|  | Reverse | TCAGCGAGTAGCGCATAGTCA |
| CTP7A1 | Forward | GGTCCTCCAGCAGAGAGCTA |
|  | Reverse | AGGAAGGAAGCATAGCGTACC |
| CYP27A1 | Forward | AGTGATGAGACAGGAGGGCA |
|  | Reverse | TCCTTGTGCGATGAAGATCCC |
| CYP7B1 | Forward | TAGCCCTCTTTCCTCCACTCATA |
|  | Reverse | GAACCGATCGAACCTAAATTCCT |
| CYP8B1 | Forward | CTGGGTCACTCCATGGCTTT |
|  | Reverse | GGAAGTCCAGCGCTTTCTCT |
| DGAT1 | Forward | TCAGATTGAGAAGCGCCTGG |
|  | Reverse | ACGGAACCCACTGGAGTGAT |
| DGAT2 | Forward | GCCGATGGGTCCAGAAGAAGTT |
|  | Reverse | CTCCAGCTTGGGGACAGTGATG |
| Dio2 | Forward | TTGGGGTAGGGAATGTTGGC |
|  | Reverse | TCCGTTTCCTCTTTCCGGTG |
| Elovl3 | Forward | GAGAAAGGATGCCACACAAC |
|  | Reverse | GAGGCTCCATCTTTCTTTCC |
| F4/80 | Forward | AGTACGATGTGGGGCTTTTG |
|  | Reverse | CCCCATCTGTACATCCCACT |
| FAS | Forward | CTGGACTCGCTCATGGGTG |
|  | Reverse | CATTTCCTGAAGTTTCCGCAG |
| FGF21 | Forward | CGAGGCTGAAAAGATGGCCT |
|  | Reverse | GCGGCAGAAGAGAGCTATAACA |
| FOXP3 | Forward | CTTAGAGAAGACAGACCCATGCTG |
|  | Reverse | GGGTTGGGCATTGGGTTCTT |
| GAPDH | Forward | TGTGTCCGTCGTGGATCTGA |
|  | Reverse | CCTGCTTCACCACCTTCTTGAT |
| GLP-1 | Forward | TCTACACCTGTTCGCAGCTC |
|  | Reverse | TCCTCATGCGCTTCTGTCTG |
| GPAT | Forward | CAGACACAGGCAGGGAATCC |
|  | Reverse | GCCTAGGTCGAAATCGCGAG |
| GRP78  (Bip) | Forward | GAGTCTGCTTCGTGTCTCCTC |
|  | Reverse | GCAGTCAGGCAGGAGTCTTAG |
| HMGCR | Forward | CTTGTGGAATGCCTTGTGATTG |
|  | Reverse | AGCCGAAGCAGCACATGAT |
| HMGCS | Forward | CCAAGACTCCCTGCAACCTC |
|  | Reverse | CCAACCGTTTCCATACCCCA |
| HSL | Forward | CGGAACTAAGTGGACGCAAGC |
|  | Reverse | TCAGACACACTCCTGCGCATA |
| IFNγ | Forward | TCAAGTGGCATAGATGTGGAAGAA |
|  | Reverse | TGGCTCTGCAGGATTTTCATG |
| IL-17 | Forward | ACTACCTCAACCGTTCCACG |
|  | Reverse | TTCCCTCCGCATTGACACAG |
| IL-1β | Forward | AGACAGGTCGCTCAGGGTCA |
|  | Reverse | AAGTGGTTGCCCATCAGAGG |
| IL-22 | Forward | GCTCAGCTCCTGTCACATCA |
|  | Reverse | CAGTTCCCCAATCGCCTTGA |
| IL-23 | Forward | AATAATGTGCCCCGTATCCAGT |
|  | Reverse | GCTCCCCTTTGAAGATGTCAG |
| IL-4 | Forward | GGCATTTTGAACGAGGTCACA |
|  | Reverse | GACGTTTGGCACATCCATCTC |
| IL-6 | Forward | TCCAGTTGCCTTCTTGGGAC |
|  | Reverse | AGTCTCCTCTCCGGACTTGT |
| Irisin  (FNDC5) | Forward | GCA TAC CCC ATC CCT GAC TC |
|  | Reverse | AGG TGG GCT TCA GTG TCT TG |
| Itgb7 | Forward | ACCCCAAGAGAGACAACAATCC |
|  | Reverse | AGTCTGCTTCCCTGGTCAGA |
| LDLR | Forward | CTGTGATCCGAGTGAGGACG |
|  | Reverse | AGTCTTCTGCTGCAACTCCG |
| LPL | Forward | GATCCGAGTGAAAGCCGGAG |
|  | Reverse | TGTTTGTCCAGTGTCAGCCAG |
| LXRα | Forward | TACGTCTCCATCAACCACCCC |
|  | Reverse | ACTTGCTCTGAATGGACGCTG |
| MCAD | Forward | AACTAAACATGGGCCAGCGA |
|  | Reverse | GAAACCTGCTCCTTCACCGA |
| MCP-1 | Forward | GTGCTGACCCCAAGAAGGAA |
|  | Reverse | GTGCTGAAGACCTTAGGGCA |
| MGL | Forward | AAACAGACTTGTGCCCGTCA |
|  | Reverse | CGACCGCTTAGGGAAGGAAA |
| MTP | Forward | ATAGCGGTCACACAACTGGC |
|  | Reverse | TTTGTAGCCCACGCTGTCTT |
| MUC13 | Forward | ATCAACCCTTCCCACCATCCT |
|  | Reverse | GCCTTTCATGGTAGCTGCTCT |
| MUC2 | Forward | GGGAGGGTGGAAGTGGCATTGT |
|  | Reverse | TGCTGGGGTTTTTGTGAATCTC |
| ND5 | Forward | AGCATTCGGAAGCATCTTTG |
|  | Reverse | TTGTGAGGACTGGAATGCTG |
| Occludin | Forward | ATGTCCGGCCGATGCTCTC |
|  | Reverse | TTTGGCTGCTCTTGGGTCTGTAT |
| PGC-1α | Forward | CCT GAA GCC GGG AGA GAA TG |
|  | Reverse | TAG CCA GCA GAG ACT GTG GA |
| PPARα | Forward | GCCGTACGCGATCAGCAT |
|  | Reverse | GTACGGTGTGTATGAAGCCATCTT |
| PPAR𝛾 | Forward | AGTGGAGACCGCCCAGG |
|  | Reverse | GCAGCAGGTTGTCTTGGATGT |
| Prdm16 | Forward | GAAGTCACAGGAGGACACGG |
|  | Reverse | CTCGCTCCTCAACACACCTC |
| SCD1 | Forward | TCAACTTCACCACGTTCTTCA |
|  | Reverse | CTCCCGTCTCCAGTTCTCTT |
| SR-B1 | Forward | GTGCCCATCATCTGCCAACT |
|  | Reverse | TGGTGACATCAGGGACTCAGA |
| SREBP1c | Forward | AGCAGCCCCTAGAACAAACAC |
|  | Reverse | CAGCAGTGAGTCTGCCTTGAT |
| SREBP2 | Forward | TGTGGAGCAGTCTCAACGTC |
|  | Reverse | GCTTTTGCCAGAGTGCTGTC |
| SRIT1 | Forward | TTGGCACCGATCCTCGAAC |
|  | Reverse | CCCAGCTCCAGTCAGAACTAT |
| TGR5 | Forward | CTT CTC TCT GTC CGC GTG TT |
|  | Reverse | GCC AGG GTT GAG GGT ACA TC |
| TNFα | Forward | GAGGCTCCAGTGAATTCGGA |
|  | Reverse | CACAAGATGCTGGGACAGTGA |
| UCP1 | Forward | CTTTGCCTCACTCAGGATTGG |
|  | Reverse | ACTGCCACACCTCCAGTCATT |
| ZO-1 | Forward | TTTTTGACAGGGGGAGTGG |
|  | Reverse | TGCTGCAGAGGTCAAAGTTCAAG |

Supplementary Table S3. Primary and secondary antibodies used in this study

| **Andibody** | **Manufacturer** | **Catalog number...** |
| --- | --- | --- |
| AMPK | Cell Signaling Technology | #2532**..** |
| β-Actin | Cell Signaling Technology | #8457**..** |
| BiP | Cell Signaling Technology | #3177**..** |
| CD36 | Cell Signaling Technology | bs-1100**..** |
| CHOP | Cell Signaling Technology | #5554**..** |
| Fatty acid synthase | Santa cruz Biotechnology | sc-20140**.** |
| FGF21 | Abcam | ab171941**.** |
| GAPDH | Cell Signaling Technology | #2118**.** |
| HSL | Cell Signaling Technology | #4107**.** |
| Occludin | Bioss Antibodies | bs-1495**.** |
| Phospho-AMPK (Thr172) | Cell Signaling Technology | #2531**.** |
| Phospho-HSL (Ser563) | Cell Signaling Technology | #4139**.** |
| SCD1 | Cell Signaling Technology | #2794**.** |
| UCP1 | Abcam | ab10983**.** |
| Anti-rabbit IgG-HRP conjugated | Cell Signaling Technology | #7074**.** |
